# Comparative proteomics of a model MCF10A-KRas^G12V^ cell line reveals a distinct molecular signature of the KRas^G12V^ cell surface

**DOI:** 10.18632/oncotarget.13566

**Published:** 2016-11-24

**Authors:** Xiaoying Ye, King C. Chan, Andrew M. Waters, Matthew Bess, Adam Harned, Bih-Rong Wei, Jadranka Loncarek, Brian T. Luke, Benjamin C. Orsburn, Bradley D. Hollinger, Robert M. Stephens, Rachel Bagni, Alex Martinko, James A. Wells, Dwight V. Nissley, Frank McCormick, Gordon Whiteley, Josip Blonder

**Affiliations:** ^1^ Cancer Research Technology Program, Frederick National Laboratory for Cancer Research, Leidos Biomedical Research, Inc., Frederick, MD 21702, USA; ^2^ Laboratory of Cancer Biology and Genetics, Center for Cancer Research, National Cancer Institute, Bethesda, MD 20892, USA; ^3^ Laboratory of Protein Dynamics and Signaling, Center for Cancer Research, National Cancer Institute, Frederick, MD 21702, USA; ^4^ Advanced Biomedical Computing Center, Frederick National Laboratory for Cancer Research, Leidos Biomedical Research, Inc., Frederick, MD 21702, USA; ^5^ Thermo Fisher Scientific, Waltham, MA 02451, USA; ^6^ Department of Pharmaceutical Chemistry, University of California, San Francisco, CA 94158-2517, USA; ^7^ UCSF Helen Diller Family Comprehensive Cancer Center, San Francisco, CA 94158-9001, USA

**Keywords:** KRas^G12V^, cell surface proteome, drug targets, proteomics, mass spectrometry

## Abstract

Oncogenic Ras mutants play a major role in the etiology of most aggressive and deadly carcinomas in humans. In spite of continuous efforts, effective pharmacological treatments targeting oncogenic Ras isoforms have not been developed. Cell-surface proteins represent top therapeutic targets primarily due to their accessibility and susceptibility to different modes of cancer therapy. To expand the treatment options of cancers driven by oncogenic Ras, new targets need to be identified and characterized at the surface of cancer cells expressing oncogenic Ras mutants. Here, we describe a mass spectrometry–based method for molecular profiling of the cell surface using KRas^G12V^ transfected MCF10A (MCF10A-KRas^G12V^) as a model cell line of constitutively activated KRas and native MCF10A cells transduced with an empty vector (EV) as control. An extensive molecular map of the KRas surface was achieved by applying, in parallel, targeted hydrazide-based cell-surface capturing technology and global shotgun membrane proteomics to identify the proteins on the KRas^G12V^ surface. This method allowed for integrated proteomic analysis that identified more than 500 cell-surface proteins found unique or upregulated on the surface of MCF10A-KRas^G12V^ cells. Multistep bioinformatic processing was employed to elucidate and prioritize targets for cross-validation. Scanning electron microscopy and phenotypic cancer cell assays revealed changes at the cell surface consistent with malignant epithelial-to-mesenchymal transformation secondary to KRas^G12V^ activation. Taken together, this dataset significantly expands the map of the KRas^G12V^ surface and uncovers potential targets involved primarily in cell motility, cellular protrusion formation, and metastasis.

## INTRODUCTION

The causal role of KRas mutants in human cancers was established three decades ago [1]. Since then, our understanding of the role of constitutively activated KRas signaling in tumorigenesis and cancer cell biology has significantly increased. However, this knowledge has not been translated into effective treatment of cancers driven by oncogenic KRas, which includes approximately 95% of pancreatic and approximately 40% of colon and lung carcinomas [2]. Notably, there are still no FDA-approved drugs capable of directly targeting oncogenic KRas. [3] Thus, direct inhibition of the oncogenic KRas and/or any of the downstream effectors remains a key goal in current cancer research.

It is well accepted that proteins residing at the cell surface of cancerous cells are easily accessible targets for both biologics (e.g., trastuzumab) [4] and/or small molecules (e.g., lapatinib) [5]. Notably, it has been shown that the differences in cell-surface protein expression are cancer- and/or cell-type specific and are reflective of their molecular signature/phenotype [6]. Hence, identification of differentially expressed proteins on the surface of cancer cells expressing oncogenic KRas may provide distinct opportunities for translation of these findings into innovative treatments [7] aimed directly at proteins unique to or exceedingly upregulated on the KRas surface.

Recent strides in cancer immunotherapy, particularly antibody-based treatments targeting proteins at the surface of cancerous cells [8, 9], further underscore the need for a comprehensive map of the KRas surface. Likewise, successes in targeting MHC-I and/or MHC-II peptides via adoptive T-cell immune-therapy [10] or virus-related proteins via prophylactic cancer vaccines [11] accentuate the urgent need for developing technologies capable of in-depth profiling of the surface of *in vitro* cultured cancer cells [12] and/or in their natural tissue microenvironment *in vivo* [13]. As a part of the NCI's RAS initiative, one project at the Frederick National Laboratory for Cancer Research (FNLCR) utilizes mass spectrometry (MS)-based proteomics to identify/characterize proteins found on the surface of cancer cells bearing oncogenic KRas. FNLCR has pioneered methods for profiling cell-surface proteins in cell lines and tissue specimens [14–18].

**Figure 1:**
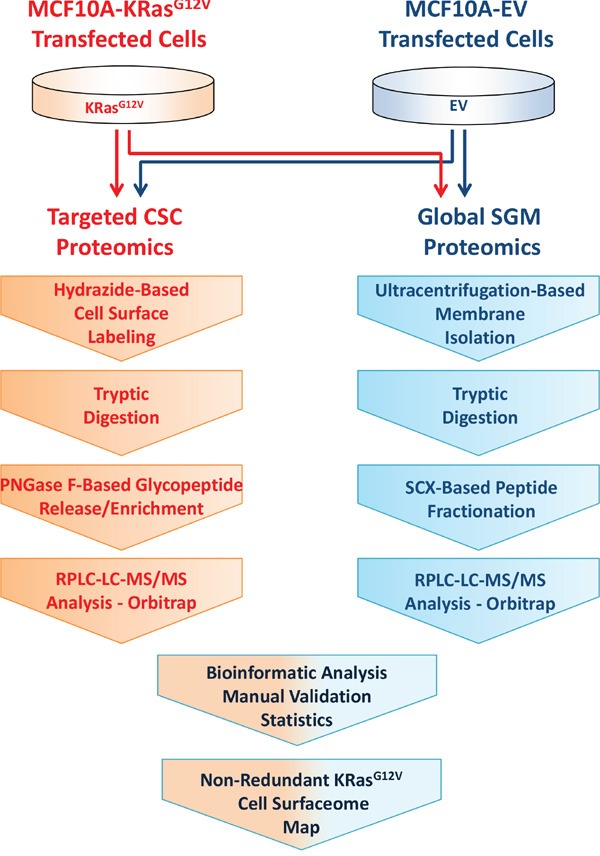
Experimental design and workflow for combined profiling of the cell surface using hydrazide based cell surface capture (CSC) technology and SCX-based shotgun membrane proteomics

Here, we describe a liquid chromatography (LC) MS-based proteomic approach for molecular phenotyping of the KRas^G12V^ surface using MCF10A-KRas^G12V^ cells as a model of oncogenic KRas transformation. To obtain a detailed map of the KRas^G12V^ surface, we applied targeted glycoprotein labeling using hydrazide-based cell surface capturing (CSC) technology [12] and global shotgun membrane (SGM) proteomics [19] to procure a broad molecular profile of the surface of MCF10A-KRas^G12V^ and MCF10A-EV cells (Figure 1).

This approach resulted in the identification of cell-surface proteins that have not previously been linked to constitutive KRas activation, along with proteins already described in the context of cancer cell lines expressing KRas mutants. Results from this investigation provide further insights into KRas-mediated tumorigenesis and offer potential novel targets residing at the surface of cells bearing oncogenic Ras. In addition, this proteomic platform permits direct quantitative measurements and large-scale investigation of signaling pathways using advanced bioinformatic tools to process data acquired at the ultimate bio-effector (i.e., protein) level, including information related to subcellular location (e.g., cell surface) and post-translational modifications (e.g., glycosylation).

## RESULTS

### Scanning electron microscopy of KRas^G12V^-transfected MCF10A cells revealed phenotypic changes typical of transformed cells

**Figure 2:**
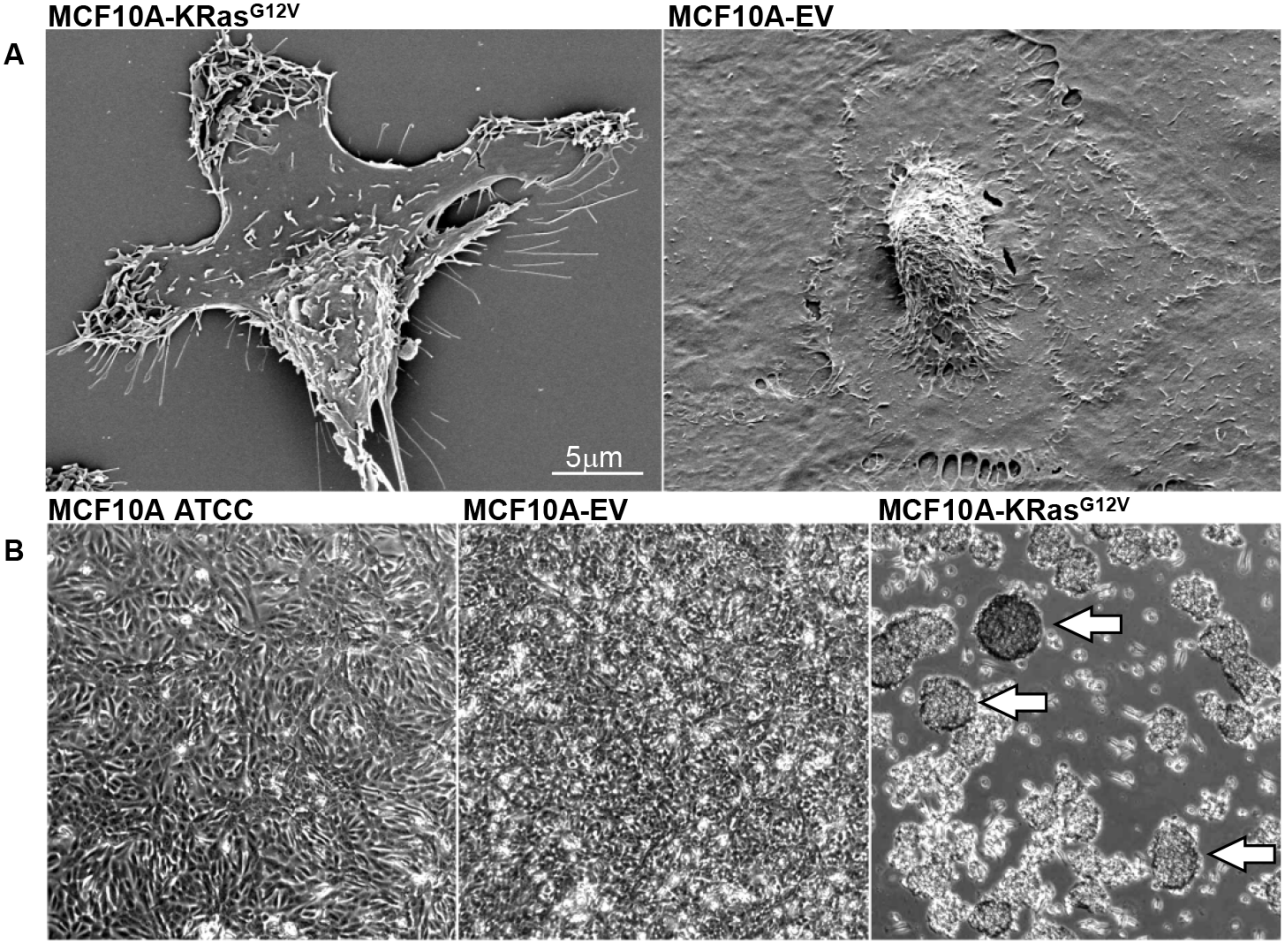
**A.** SEM images showing surface morphology of transformed MCF10A-KRas^G12V^ and control MCF10A-EV cells. **B.** Phase microscopy images of non-manipulated MCF10A-ATCC cells, control MCF10A-EV cells, and MCF10A-KRas^G12V^ cells in culture. Arrows pointing to sphere formation.

At the outset, we carried out a comparative scanning electron microscopy (SEM) analysis of MCF10A-KRas^G12V^ and control MCF10A cells virally transduced with empty vector (MCF10A-EV) to examine the extent and nature of changes in cell-surface morphology secondary to the oncogenic KRas activation. SEM has been frequently used to study the morphology of the surface of cultured cells [20, 21]. The SEM analysis revealed altered morphology of the MCF10A-KRas^G12V^ cells characterized by spindle-shaped bodies and multiple cell-surface protrusions that are consistent with cellular protrusions formation (Figure 2A). These findings support increased mobility/invasion capabilities and are suggestive of epithelial-to-mesenchymal transformation (EMT) [22]. On the contrary, the surface of control MCF10A-EV cells showed flat “cobblestoned” surfaces and exhibited a globule-shaped nucleus visible in the cell center, features of well-differentiated non-malignant epithelial cells (Figure 2A) [22]. In addition, we observed that MCF10A-KRas^G12V^ cells form spheres (Figure 2B) if grown in high densities. This feature was absent during the culture of MCF10A-EV and parental MCF10A-ATCC cells, which formed a monolayer (Figure 2B).

### Phenotypic cancer cell assays disclosed features consistent with EMT-like driven malignant transformation of MCF10A-KRas^G12V^ cells

Next, we carried out phenotypic cell assays to investigate changes secondary to KRas^G12V^ transfection of epithelial MCF10A cells. Phenotypic cancer cell assay screens are commonly used in the process of cancer drug discovery [23]. In comparison to MCF10A-EV cells, the KRas^G12V^ transfected cells showed an increase in invasion, migration, and anchorage independent growth (Supplementary Figure S1A–S1C). Amplified migration is consistently observed in malignantly transformed cells, whereas positive invasion and anchorage independence assays are suggestive of an acquired ability of MCF10A-KRas^G12V^ cells to invade and metastasize. Taken together, the results of the SEM and phenotypic cancer assays authenticate the transition of the regular epithelial MCF10A-EV phenotype towards the malignantly transformed EMT-like MCF10A-KRas^G12V^ phenotype, secondary to constitutive activation of the oncogenic KRas.

### Profiling the cell surface glyco-proteome of the MCF10A-KRas^G12V^ cells using MS-based cell surface proteomics

To identify and characterize protein species unique and/or upregulated on the surface of MCF10A cells bearing constitutively activated KRas protein, we developed a strategy that combines two orthogonal proteomic methods. One is hydrazide-based CSC technology targeting cell surface glycoproteins [12], and another is global SGM proteomics for global profiling of membrane proteins [19]. The experimental design and workflow is depicted in Figure 1.

First, we applied the CSC technology [24] to capture the differences between the surface glyco-proteome of MCF10A-KRas^G12V^ and MCF10A-EV cells. To ensure reliability and increase surfaceome coverage, the samples were prepared on two independent occasions (i.e., two biological replicates). Each sample was injected three times (i.e., three technical replicates) into the high-resolution/accuracy hybrid MS. The liquid chromatography mass-spectrometric (LC-MS) analysis resulted in a total of 148 and 122 glycoproteins identified (Supplementary Figure S2A) on the surface of MCF10A-KRas^G12V^ and MCF10A-EV cells, respectively, matching a stringent peptide false discovery rate of ≤0.01%, as calculated by the Percolator algorithm (Supplementary Tables S1A–S2A) [25]. All proteins were identified exclusively by affinity-captured glyco-peptides (Supplementary Tables S1B–S2B), displaying the PNGase F–induced asparagine deamidation shift of 0.98 Da and obligatory N-glycosylation amino-acid Nx(S/T) sequence motif.

To ascertain and validate the subcellular location of the identified proteins, we compared our data to the list of 1,492 human proteins catalogued in the Cell Surface Protein Atlas (CSPA) (http://wlab.ethz.ch/cspa) The CSPA provides the evidence for experimentally identified cell-surface proteins from 41 distinct human cell types [26]. This comparison revealed that 145 of 148 (98%) and 118 of 122 (97%) proteins identified on MCF10A-KRas^G12V^ and MCF10A-EV cells, respectively, were annotated as the human cell surface proteins in CSPA (Supplementary Tables S1A–S2A). These results are indicative of the high efficiency of the present methodology to target and enrich for protein species residing on the surface of cultured cells.

Similarly, the search against the cluster of differentiation (CD) cell-surface molecules (HUGO database containing 386 entries) showed that 44 (30.3%) and 36 (30.2%) glycoproteins detected on the surface of MCF10A-KRas^G12V^ and MCF10A-EV cells, respectively, were recognized CD molecules (Supplementary Tables S1A–S2A) [27]. This represents a 15-fold increase in enrichment of CD molecules in our dataset when compared to the 2% fraction of CD molecules contained within the entire non-redundant SwissProt human proteome database (v57/15), which contains a total of 20,193 protein entries.

### Classification of glycoproteins identified on the surface of MCF10A-KRas^G12V^ and MCF10A-EV cells

Both cancerous and normal cells recognize and react to environmental signals via cell-surface proteins. We employed the PANTHER classification system [28] to compare functional capacities, categorize protein classes, and investigate the enrichment of signaling pathways within the complement of identified glycoproteins on the surface of MCF10A-KRas^G12V^ and MCF10A-EV cells. This analysis revealed comparable sharing of functional groups between MCF10A-KRas^G12V^ and MCF10A-EV cells. The most enriched functional groups included receptor activity, binding, and carrier activity (Supplementary Figure S2B–S2C). The most prevalent protein classes were receptors, transporters, and adhesion molecules, showing a slightly increased identification enrichment rate on the surface of MCF10A-KRas^G12V^ cells (Supplementary Figure S3A). Furthermore, PANTHER pathway analysis showed an increased identification/enrichment rate of glycoproteins implicated in integrin, [29] cadherin, [30] Wnt, [31] MAPkinase, [32] EGF, [33] chemokine, and cytokine signaling [34] pathways on the surface of MCF10A-KRas^G12V^ cells (Supplementary Figure S3B).

### Subtractive and comparative proteomics exposed substantial differences between the surface glyco-proteomes of MCF10A-KRas^G12V^ and MCF10A-EV cells

While results of the global allocation of molecular functions were similar for MCF10A-KRas^G12V^ and MCF10A-EV cells, the initial comparison of protein species within corresponding biological function and protein groups revealed remarkable differences between the gene products identified on the surface of MCF10A-KRas^G12V^ and MCF10A-EV cells. Using label-free spectral counting–based relative quantitation [35] and subtractive proteomics [17] to reveal all of the gene products upregulated or uniquely expressed on the MCF10A-KRas^G12V^ surface, we identified a subset of 86 glycoproteins on the surface of both cell lines (Supplementary Figure S2A). Importantly, a total of 62 glycoproteins was identified solely on the surface of MCF10A-KRas^G12V^ cells (Supplementary Table S3A), and 46 were found significantly upregulated on the surface of MCF10A-KRas^G12V^ cells (Supplementary Table S3B).

### Pathway analysis revealed cellular movement, cancer signaling, and embryonic development as the three most enriched functional glycoprotein networks on the surface of MCF10A-KRas^G12V^ cells

To further investigate the biological relevance of the glyco-proteomic results and prioritize targets for cross-validation using subtractive and comparative proteomics, a subset of proteins detected exclusively and/or found upregulated on the MCF10A-KRas^G12V^ surface was subjected to the Ingenuity^®^ Pathway Analysis (IPA^®^) [QIAGEN Redwood City, www.qiagen.com/ingenuity]. The IPA^®^ has been extensively used by the scientific community for analysis of proteomic, genomic, and metabolomic data. The IPA^®^ “Network Analysis” revealed cellular movement, cancer and cellular movement signaling, and embryonic development as the three top functional networks in terms of statistical significance and number of interacting surface proteins (Supplementary Table S4).

The cellular movement network (Supplementary Figure S4) showed that the majority of proteins in this cluster interact with the serine/threonine kinase AKT signaling node. Out of 18 identified molecules depicted in this network, a total of 10 N-glycosylated upregulated gene-products (ALCAM, CDCP1, CDH2, ITGAV, MCAM, NEO1, PLAU, PTPRJ, PTPRM, and TIMP1) were associated with Ras signaling [36–44]. However, the only gene product in this network that has been explicitly implicated in the movement of cancer cells expressing specifically oncogenic KRas mutants is CDCP1 [37, 45]. The remaining eight gene products identified solely (CDH4, LAMC1, PTK7) or found upregulated (DSG2, ITGB5, LRP1, LRRC8A, and PTPRG) on the MCF10A-KRas^G12V^ surface had no known connection with oncogenic KRas signaling established in the literature.

The cancer signaling and cellular movement network (Supplementary Figure S5) depicts RAS as the most prominent signaling node along with RAC and PI3K. This underscores the high biological content and relevance of gene products found exclusively and/or upregulated on the MCF10A-KRas^G12V^ surface. Out of 15 identified molecules depicted in this network, eight gene products had a common connection with the Ras pathway signaling established in the literature, of which four were detected solely (EPHA2, IGF1R, IGF2R, and MME) [46–49] on the MCF10A-KRas^G12V^ surface, while the other four (BSG (CD147), CD44, ITGB4, and M6PR) [50–53] were found upregulated on the MCF10A-KRas^G12V^ surface. Of these, CD147, CD44, EPHA2, IGF1R, and ITGB4 had direct involvement in the cell mobility reported in the literature in the context of KRas-driven malignant transformation [46, 47, 50–52]. The remaining seven molecules had no connection with oncogenic KRas signaling in the cancer cell lines established in the literature. These include CADM3 and EFNB2 detected solely on the MCF10A-KRas^G12V^ surface as well as five upregulated surface proteins (ADAM10, ADAM15, PLXNB2, PRNP, and PROCR).

The embryonic development network (Supplementary Figure S6) depicts ERK (signaling molecule of the Ras/Raf/MEK/ERK pathway) as the most prominent signaling node. It is widely accepted that genes involved in embryonic development are often aberrantly activated during tumorigenesis [54]. Conceivably, the pathways controlling rapid, well-regulated cell growth and migration during embryogenesis are frequently dysregulated/hijacked during uncontrolled tumor growth and metastasis [55]. Hence, highly controlled ERK signaling during normal embryogenesis [56] is habitually dysregulated and critical for initiation and development of many human cancers [57]. Out of 13 molecules identified in this network, only four gene products, including GPC1 and IL6ST, detected solely on the MCF10A-KRas^G12V^ surface, along with F3 and SLC2A1, found upregulated on the MCF10A-KRas^G12V^ surface, had known direct association with embryogenesis and oncogenic KRas activation established in the literature [58–61]. The remaining nine surface proteins identified solely (ANTXR1, AG1, DCBLD2, EFNB1, EMB, OSMR, SLC1A5) or found upregulated (ATP1B3 and ITGB1) on the MCF10A surface had no direct association with embryonic development signaling in the context of KRas mutant signaling in cancer cell lines.

Next, we carried out the IPA^®^ “Upstream Analysis” to elucidate the upstream regulators that can explain the changes in glycoprotein regulation captured by targeted CSC proteomics, as well as to further assess the relevance of the acquired data. It is conceivable that the KRas protein was not identified by CSC technology, which exclusively targets glycoproteins located on the outer leaf of the plasma membrane. However, the upstream IPA^®^ analysis revealed/predicted KRas as an activated upstream regulator in MCF10A-KRas^G12V^ cells (Supplementary Figure S7A). The outcome of upstream IPA^®^ analysis is based solely on the observed changes in regulation of KRas downstream targets, including gene products of AREG, CDH2, IGF1R, and MCAM detected solely on the KRas^G12V^ surface and ANPEP, CD147, CD44, F3, NT5E, and SLC3A2 found upregulated on the surface of MCF10A-KRas^G12V^ cells using CSC technology (Supplementary Figure S7A). This finding further validates the biological significance/utility of results obtained by CSC proteomics.

Subsequent IPA^®^ “Regulator Effects Analysis” revealed TGF-β as the principal regulator of cellular protrusions formation via downstream activation/targeting of N-glycosylated ANGLPTL4, CDH2, FN1, and PLAU that were detected solely on the KRas^G12V^ surface, along with ITGB1 that was found upregulated (Supplementary Figure S7B). The regulator effects analysis provides insight into the causes and effects of differentially expressed genes or proteins in a given dataset. It helps explain how predicted activated and/or inhibited upstream regulators might cause downstream increases or decreases in phenotypic or functional outcomes. The overlap of results acquired via upstream regulator networks and downstream effects networks may facilitate the development of causal hypotheses in the form of directionally coherent networks generated from their merger [62].

### Cross-validation of selected differentially regulated surface glycoproteins

We used immunofluorescence (IF) analysis, conventional microscopy, structured illumination microscopy (SIM), and western blotting (WB) to cross-validate the subcellular location and expression level of selected surface targets identified by LC-MS. The prioritization and selection of targets was a multistep process driven by the results of cellular component analysis, subtractive/comparative proteomics, protein classification, biological categorization, pathway analysis, manual validation/selection of corresponding MS^2^ spectra, and antibody availability/accessibility. Accordingly, the N-glycosylated gene products of ANTXR1, CDCP1, CDH4, CD147, IGF1R, MCAM, PROCR, and PTPRJ depicted in the three top IPA^®^ signaling networks were selected for cross-validation.

**Figure 3:**
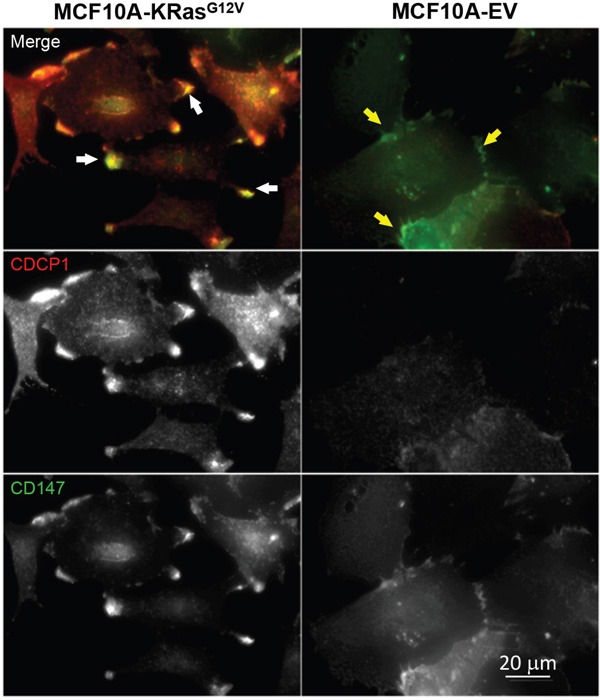
Localization of CD147 and CDCP1 in MCF10A-KRas^G12V^ cells Maximum intensity projection images of representative cells in population. In cells expressing oncogenic KRasG12V, both proteins are enriched on the cell surface, especially within cellular protrusions (white arrows). In control cells (EV), both proteins are distributed in the cytoplasm, with some accumulation to the sites of cell-cell contacts (yellow arrows). Immunolabeling was carried out using commercially available antibodies.

Based on findings obtained by SEM (e.g., cellular protrusions formation) and phenotypic cancer assays (e.g., increased mobility, invasion, and anchorage independent growth), we first selected basigin (CD147) and CUB domain-containing protein (CDCP1) for cross-validation in connection with their previously established roles in cell motility, invasion, cellular protrusions formation, and metastasis in the context of the oncogenic KRas-driven transformation [50, 63]. CD147 and CDCP1 were found significantly upregulated on the MCF10A-KRas^G12V^ surface by LC-MS and were depicted in the two top IPA^®^ networks (cell motility and cancer). Following the cell surface immune-labeling using commercially available antibodies, the comparative microscopy analysis of MCF10A-KRas^G12V^ and MCF10A-EV cells confirmed the upregulation of CD147 and CDCP1 on the MCF10A-KRas^G12V^ surface, with the strongest signal localized at the very top of the cellular protrusions surface (Figure 3).

**Figure 4:**
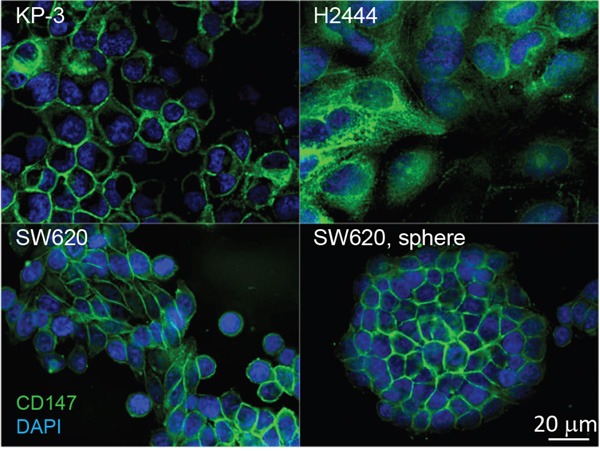
Distribution of CD147 in cancer cell lines expressing oncogenic KRas^G12V^ mutants endogenously, pancreatic (KP-3), lung (H2444), colon (SW620), and SW620 spheroid Cells were immunolabeled for CD147 using commercially available antibodies and DNA was labeled with DAPI. One 200 nm section through the middle of the cells is presented to illustrate localization of CD147 on the cell surface.

To further investigate the localization of CD147 in cancer cell lines expressing KRas^G12V^ endogenously, we carried out IF analysis targeting CD147 on the surface of pancreatic (KP3), lung (H2444), and colon cancer (SW620) cell lines. The microscopy analysis revealed a positive CD147 signal on the surface of all selected cell lines (Figure 4), suggesting a common role of CD147 in motility, invasion, and metastasis of cancer cells expressing the KRas^G12V^ mutant, regardless of their tissue of origin. Notably, the IF analysis of spheres observed during the culture of SW620 revealed explicit CD147 expression on the surface of sphere-forming cells (Figure 4). This finding is in agreement with results of previous investigations, which point toward a critical role of CD147 in the biology of colorectal cancer stem cells (CSCs) [64]. The results also expose CD147 as a potential CSC-specific target in the context of the metastatic disease of KRas^G12V^-driven cancers [65] and are indicative of the oncogenic KRas-impelled stemness and therapeutic resistance [29].

**Figure 5:**
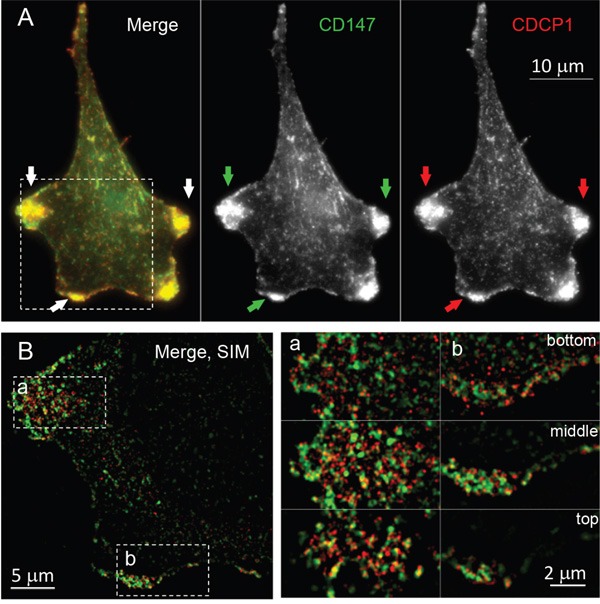
Colocalization of CD147 and CDCP1 on the surface of MCF10A-KRas^G12V^ cells **A.** Maximum intensity projection image of a representative MCF10A-KRasG12V cell, immunolabeled for CD147 and CDCP1 using commercially available antibodies. Both proteins are enriched on the leading edges of the cell, as marked by arrows. **B.** Structured Illumination Microscopy (SIM) of the same cell reveals that two proteins closely associate and partially co-localize on the leading edges and membrane ruffles. One (middle) section of the cell is present on the left. Bottom, middle, and the top section of two areas (a and b) are enlarged on the right.

**Figure 6:**
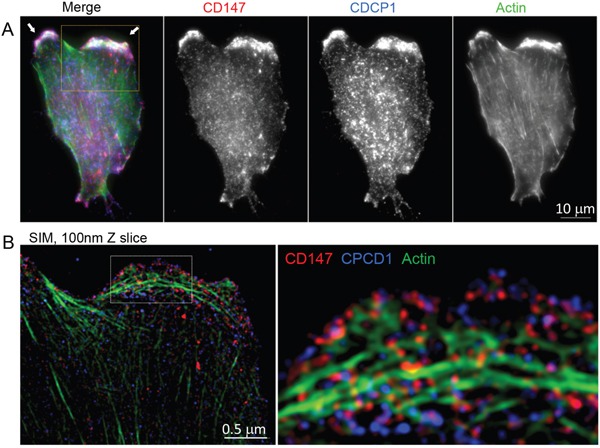
Localization of CD147, CDCP1 and actin in MCF10A-KRas^G12V^ cells **A.** Maximum intensity projection image of a representative MCF10A-KR cell, immunolabeled for BSG and CDCP1. Actin is visualized by phalloidin. All three proteins are enriched on the surface of cellular protrusions. **B.** Structured Illumination Microscopy (SIM) analysis of the part of the same cell outlined by the yellow square. The part of the cell outlined with a white dashed-line square is further enlarged. This Figure is associated with Supplementary Movie 1.

**Figure 7:**
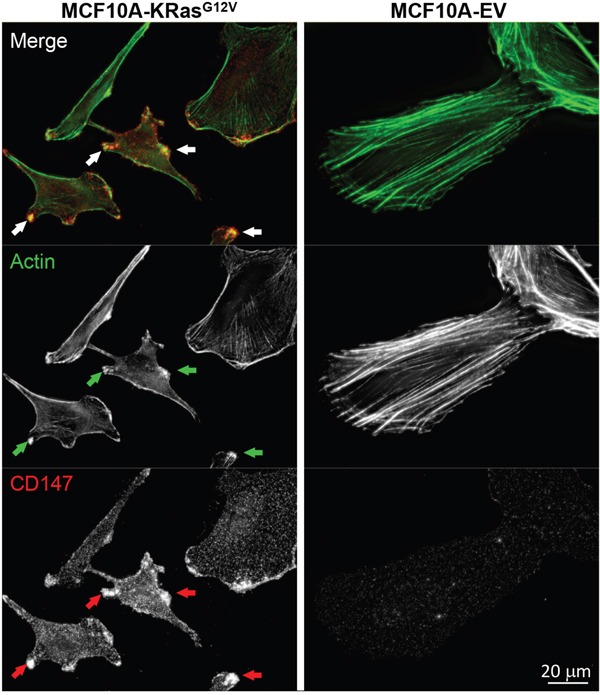
Redistribution of actin in MCF10A-KRas^G12V^ cells Maximum intensity projection images of representative cells in population. Cells were immunolabeled for CD147. Phalloidin labeling was used to visualize actin cytoskeleton. In cells expressing KRas^G12V^ actin cytoskeleton is redistributed. Bright actin foci co-localize with the sites of CD147 accumulation on cellular surface protrusions (arrows).

Based on the similarity of the subcellular locations of CD147 and CDCP1 within cellular protrusions of MCF10A-KRas^G12V^ cells depicted in Figure 3, we examined the proximity and possible co-localization of these two proteins on the surface of cellular protrusions. Indeed, SIM revealed numerous co-localized or adjacent CD147 and CDCP1 signals on the surface of MCF10A-KRas^G12V^ cells (Figure 5). In addition, CDC147 and CDCP1 signals were often detected in close vicinity of actin filaments (Figure 6). Interestingly, SIM analysis also revealed differences in actin organization between control and transformed cells (Figure 7). Actin was depicted as heavily enriched within cellular protrusions of MCF10A-KRas^G12V^ cells. This is in agreement with the active role of KRas signaling in actin nucleation and cancer cells invasion, [66–68] and critical role of actin in formation of cellular protrusions [69]. While others have demonstrated independent roles of CD147 (cellular protrusions formation, cell motility) [50] and CDCP1 (invasion, anchorage-independent growth) [63], this is the first report explicitly showing their wide-ranging co-localization on the KRas^G12V^ surface (Figures 6–7).

**Figure 8:**
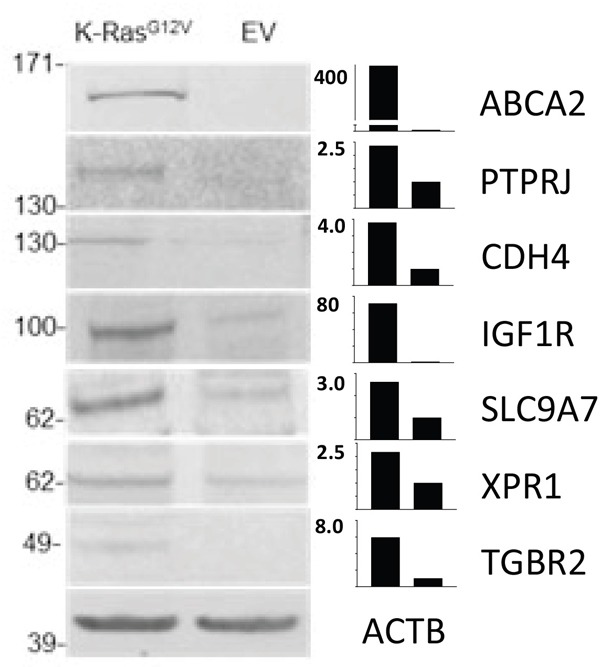
Comparative WB analyses carried out on crude membrane preparations from MCF10-KRas^G12V^ and MCF10A-EV cells Cropped images show WB analyses using commercially available antibodies against gene products of ABCA2, PTPRJ, CDH4, IGF1R, SLC9A7, XPR1, and TGFBR2 using actin (ACTB) as control. Bar graphs show the relative expression levels of the target proteins in KRas^G12V^ to MCF10A-EV cells.

**Figure 9:**
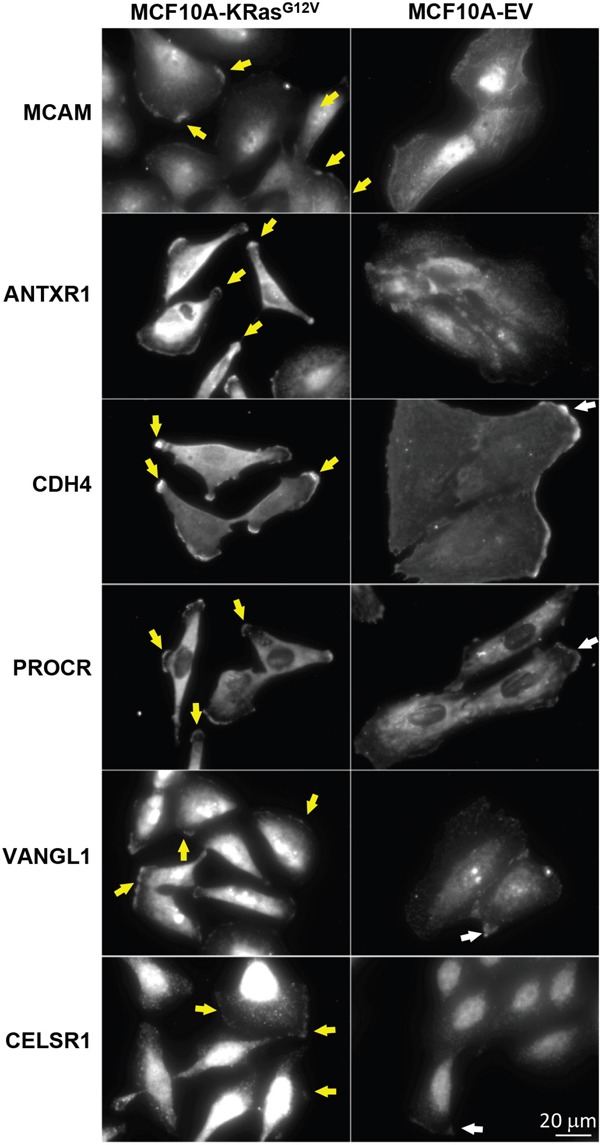
Comparative microscopy analysis of immunolabeled MCAM, ANTXR1, CDH4, PROCR, VANGL1, and CELSR1 gene products using commercially available antibodies in MCF10-KRas^G12V^ and MCF10A-EV cells Maximum intensity projection images of representative cells show upregulation and/or unique expression on the cell surface of KRas^G12V^ cells, especially within invadopodia (yellow arrows). In control cells (EV) respective proteins are distributed in the cytoplasm, with some accumulation to the sites of cell-cell contacts (white arrows).

Subsequently, we cross-validated insulin-like growth factor 1 receptor (IGF1R) and receptor-type tyrosine-protein phosphatase eta (PTPRJ) via WB analysis (Figure 8), along with surface glycoprotein MUC18 (MCAM) via IF (Figure 9). These molecules have already been proposed as viable targets in the context of immunotherapeutic approaches for KRas-driven cancers [70–73]. Positive cross-validation of KRas surface targets (i.e., CD147, CDCP1, IGF1R, MCAM, and PTPRJ) previously discovered using non-proteomic approaches [42, 47, 50, 63, 73, 74] are indicative of the utility of the present technology for effective and confident profiling of the KRas surface using present MS-based proteomic approach.

Next, we cross-validated selected gene products that had no explicit connection with the oncogenic KRas signaling established in the literature, using IF and/or WB. These include anthrax toxin receptor (ANTXR1), cadherin-4 (CDH4), and endothelial protein C receptor (PROCR) (Supplementary Table S4A). The cross validation findings were consistent with results obtained by LC-MS (Figures 8–9).

ANTXR1 is a single-pass cell-surface glycoprotein originally identified in the tumor-infiltrating vasculature [75]. It has been implicated in the regulation of tumor neo-vasculature [76] and reported to be highly expressed during normal embryonic development [77]. The IF analysis (Figure 9) unambiguously showed ANTXR1 expression on the MCF10A-KRas^G12V^ surface as well as the lack of the IF signal from the surface of MCF10A-EV cells. While the use of anti-ANTXR1 antibodies targeting the tumor's neo-vasculature (stroma) has been investigated previously, [78] this is the first report to suggest the presence of ANTXR1 on the surface of malignantly transformed epithelial cells (parenchyma) in the context of the oncogenic KRas-driven tumorigenesis.

Cadherin-4 (CDH4) belongs to a superfamily of transmembrane surface proteins. Cadherin switching is an essential event in the process of malignant transformation [79]. While the role of CDH4 in proliferation, cellular motility, invasion, and metastasis in a Rho GTPase-dependent manner has been described previously in the context of gastric and nasopharyngeal carcinoma, [80] there are no reports linking CDH4 with oncogenic KRas signaling. The results of WB (Figure 8) and IF (Figure 9) analyses are consistent with marked upregulation of CDH4 on the surface of MCF10A-KRas^G12V^ cells.

Endothelial protein C receptor (PROCR) belongs to the protein C pathway that controls blood coagulation as well as cancer progression [81]. A recent investigation describes the tumor-initiating properties of PROCR detected in cancer stem-like cells in the context of aggressive invasive/metastatic carcinomas, and this is the first report showing the upregulation of PROCR on the surface of cells expressing oncogenic KRas^G12V^ (Figure 9).

### Expanding the map of the MCF10A-KRas^G12V^ surface using SGM proteomics

To further expand the MCF10A-KRas^G12V^ surface map, we employed SGM proteomics as previously described [19, 82]. This strategy has been proven effective in proteomic profiling of complex membrane protein mixtures [82, 83]. A total of 12 pooled SCX peptide fractions from MCF10A-KRas^G12V^ and MCF10A-EV membrane preparations were collected and injected twice (i.e., two technical replicates) to carry out high resolution/accuracy LC-MS analysis.

This analysis resulted in the identification of a total of 4,869 and 4,287 proteins in crude membrane preparations of MCF10A-KRas^G12V^and MCF10A-EV cells (Supplementary Tables S5A–S6A), from a total of 27,645 and 30,603 peptides (Supplementary Tables S5B–S6B), respectively. Bioinformatic processing of initial SGM data using PSORT and TMHMM membrane prediction algorithms revealed a total of 1,338 (27.5%) and 1,086 (25.3%) membrane proteins identified in MCF10A-KRas^G12V^ and MCF10A-EV cells, respectively (Supplementary Tables S5A–6A). The membrane protein identification rate is in agreement with the predicted proportion of membrane proteins in the human proteome. Of these, a total of 763 and 605 protein-species (Supplementary Figure S8A) were mapped using the IPA^®^ knowledge database (Supplementary Tables S5A–6A) as genuine cell-surface proteins in MCF10A-KRas^G12V^ and MCF10A-EV cells, respectively.

The search against the CD surface complement (HUGO database containing 386 entries) showed that 76 (9.97%) and 60 (9.93%) gene products identified on the surface of MCF10A-KRas^G12V^ and MCF10A-EV cells using global SGM proteomics were annotated as CD molecules (Supplementary Tables S5A–6A) [27]. This represents a fivefold enrichment when compared with the 2% fraction of CD molecules contained within the entire human proteome. The CD molecules enrichment rate obtained by global SGM proteomics was lower than the enrichment rate obtained by CSC technology. Nonetheless, SGM makes a significant addition to the complement of CD molecules already identified by targeted CSC technology on the surface of MCF10A-KRas^G12V^ and MCF10A-EV cells.

### Classification of proteins identified on the cell surface of MCF10A-KRas^G12V^ and MCF10A-EV cells using SGM proteomics

To compare protein functions and protein classes and examine signaling pathways of protein complements identified via SGM proteomics on the surface of MCF10A-KRas^G12V^ and MCF10A-EV cells, we employed the PANTHER classification system [28]. This analysis revealed a comparable distribution of functional groups between MCF10A-KRas^G12V^ and MCF10A-EV cells. The most representative functional groups included binding activity, catalytic activity, receptor activity, and transporter activity (Supplementary Figure S8B–S8C). The most prevalent protein classes were receptors, transporters, and enzyme modulators showing a slightly increased identification rate on the surface of MCF10A-KRas^G12V^ cells (Supplementary Figure S9A). Furthermore, the PANTHER pathway analysis showed an increased identification rate of cell-surface proteins implicated in integrin, [29] chemokine/cytokine, [34] Wnt, [31] and angiogenesis [84] signaling pathways (Supplementary Figure S9B). Although general in nature, the results of the pathway classification were in agreement with the role of these pathways in KRas-driven malignant transformation.

### Differential SGM proteomics exposes differences between the surfaces of MCF10A-KRas^G12V^ and MCF10A-EV cells

An initial examination of the cell-surface proteins identified via SGM proteomics revealed significant differences between the cell-surface protein complements identified on the MCF10A-KRas^G12V^ and on the MCF10A-EV cells. A subsequent subtractive/comparative proteomic analysis of a total of 763 and 605 protein species identified on the surface of MCF10A-KRas^G12V^ and MCF10A-EV cells revealed a subset of 301 proteins detected solely on the surface of MCF10A-KRas^G12V^ cells (Supplementary Table S7A). Out of 462 surface proteins found differentially regulated on the surface of both cell lines, a total of 168 proteins were found significantly upregulated on the surface of MCF10A-KRas^G12V^ cells (Supplementary Table S7B).

A comparison against the human CSPA revealed that a total of 268 (35.2%) and 191 (31.6%) proteins identified by SGM proteomics on the surface of MCF10A-KRas^G12V^ and MCF10A-EV cells, respectively, are N-glycosylated protein species. These results indicate that SGM proteomics have a significant capability to expand the coverage of the surface map, including glycosylated protein species (Supplementary Tables S6A–6B).

### Pathway analysis and cross-validation of selected cell-surface targets identified via SGM proteomics

To assess the biological relevance of KRas-regulated proteins and prioritize cross-validation targets, we carried out the IPA^®^ network analysis of a subset of cell-surface proteins found upregulated or identified solely on the MCF10A-KRas^G12V^ surface using SGM proteomics. Comparable to the results of glyco-proteomic analysis, the IPA^®^ network analysis of the SGM data exposed cellular movement and cancer signaling, drug metabolism, cellular movement and invasion, formation of cellular protrusions, and cancer as significantly enriched networks (Supplementary Table S4B).

Based on the IPA^®^ network analysis/filtering and availability of commercial antibodies, the sodium/hydrogen exchanger 7 (SLC9A7) from the cancer and cellular movement network (Supplementary Figure S10), the ATP-binding cassette sub-family A member 2 (ABCA2) from the drug metabolism network (Supplementary Figure S11), the TGF-beta receptor type-2 (TGFBR2) from the cellular movement and invasion network (Supplementary Figure S12), and the cadherin EGF LAG seven-pass G-type receptor 1 (CELSR1) from the formation of cellular protrusions network (Supplementary Figure S13) were selected for cross-validation from the pool of targets identified solely on the MCF10A-KRas^G12V^ surface. From a subset of gene products found upregulated on the surface of MCF10A-KRas^G12V^, the xenotropic and polytropic retrovirus receptor 1 (XPR1) from the formation of cellular protrusions network (Supplementary Figure S13) and the vang-like protein 1 (VANGL1) from the cancer network (Supplementary Figure S14) were selected for cross-validation. Of these, gene products of ABCA2, CELSR1, SLC9A7, XPR1, and VANGL1 had no connection with the oncogenic KRas^G12V^ signaling established in the literature. Only TGFBR2 had a link with the oncogenic KRas signaling established in the literature [74].

While ABCA2, CELSR1, and TGFBR2 were annotated as N-glycosylated surface molecules in human CSPA, the remaining targets, including SLC9A7, VANGL1, and XPR1, were not found in human CSPA and are not predicted to be post-translationally glycosylated by the non-redundant UniProtKB/SwisProt database annotations. The results of subsequent WB analyses for ABCA2, SLC9A7, TGFBR2, and XPR1 (Figure 8), along with the results of IF analyses of CELSR1 and VANGL1 (Figure 9), were in agreement with LC-MS results.

### Defining a non-redundant surface map of MCF10A-KRas^G12V^ cells via combined CSC and SGM proteomics

Finally, we generated a non-redundant surface MCF10A-KRas^G12V^ map compiled from the results of a combined application of targeted CSC glyco-proteomics and global SGM proteomics. This analysis revealed a complement of 504 non-redundant surface protein species detected uniquely (308) or found significantly upregulated (195) on the KRas surface (Supplementary Table S8A–B). Out of 308 non-redundant protein species identified solely on the MCF10A-KRas^G12V^ surface, a total of 177 (57.4%) molecules were authentic glycoproteins catalogued in human CSPA. The remainder of the 131 (42.6%) molecules were annotated in the IPA^®^ knowledge database as genuine cell-surface proteins identified by SGM proteomics (Supplementary Table S8A). Correspondingly, amongst 195 non-redundant cell-surface proteins found significantly upregulated on the KRas^G12V^ surface using spectral counting–based relative quantitation, a total of 89 (45.6%) molecules were cell-surface glycoproteins catalogued in CSPA. The remaining 106 (54.4%) molecules were annotated as authentic cell-surface proteins in the IPA^®^ knowledge database, identified by global SGM proteomics (Supplementary Table S8B).

Subsequent IPA^®^ canonical pathway analysis of cell-surface proteins found upregulated or identified solely on the MCF10A-KRas^G12V^ surface revealed statistically significant enrichment and activation of pancreatic adenocarcinoma signaling, non-small cell lung cancer signaling, colorectal cancer metastasis signaling, and actin nucleation by ARP-WASP complex pathways (Supplementary Table S9). These results are in agreement with the pivotal role of KRas mutants in the biology of pancreatic, lung, and colon carcinomas [3] and the role of actin nucleation in cellular protrusions formation [69]. The same analysis revealed axonal guidance signaling as the top-ranked pathway by IPA^®^ in terms of statistical significance and number of identified molecules (Supplementary Table S9). Notably, the alteration of the axon guidance signaling pathway was the major finding of genomic analysis that relied on exome sequencing and copy number analysis to profile a total of 142 prospectively collected pancreatic (stage I and II) ductal adenocarcinomas [85].

While the selection/prioritization of cell-surface targets in present investigation was a multistep process driven primarily by literature-based bioinformatic processing (IPA^®^ analysis), statistics, manual validation of raw MS data, and commercial antibody quality/availability in the context of examining/demonstrating the feasibility (i.e., proof of principle study) of present methodology, any of the cell-surface proteins uncovered in this study may be further investigated as potential/viable targets and/or cell-surface markers.

### Combined application of CSC technology and SGM proteomics recapitulates the KRas^G12V^ phenotype and validates the MCF10A-KRas^G12V^ Cell Line Model

Due to the accessibility of integral and membrane-associated proteins on both sides of the cell membrane via combined CSC technology [12, 26] and SGM proteomics [13, 16, 83], we sought to examine the extent of the Ras pathway coverage and assess the utility of MCF10A-KRas^G12V^ as a model cell line. Using the present methodology, out of the 227 molecules contained in the latest Ras (Ras 2.0) pathway's draw, accessible at the FNLC-Ras Initiative website, a total of 158 gene products were identified. It resulted in 70% of the Ras 2.0 pathway coverage, corresponding to 84 and 76 Ras pathway proteins identified in MCF10A-KRas^G12V^ and MCF10A-EV cells, respectively (Supplementary Table S10). Subsequent IPA^® “^Canonical Pathway Analysis” confirmed activation of the ERK/MAPK signaling in MCF10A-KRas^G12V^ cells (Supplementary Figure S15). As expected, the spectral counting–based quantitation exposed KRas as the most abundant Ras isoform identified in the membrane fraction of MCF10A-KRas^G12V^ cells, and SGM proteomics showed a more than 40-fold upregulation rate in comparison to wild-type KRas expression in MCF10A-EV cells. (Supplementary Table S8B). Interestingly, significant upregulation of the NRas and HRas isoforms was also observed in the membrane fraction of MCF10A-KRas^G12V^. All Ras isoforms were identified by isoform-specific peptides (Supplementary Tables S5B–6B). These findings were in agreement with the results of an elegant study reported by the Bar-Sagi group, which showed a similar outcome suggestive of the dependence of KRas mutants on wild-type HRas and NRas isoforms [86–88]. Next, we carried out WB analysis of the corresponding membrane fraction using commercial antibodies against KRas, NRas, and SOS2 to validate the LC-MS findings. Indeed, the results of WB analyses confirmed upregulation of KRas, NRas, and SOS2 in the membrane fraction of MCF10A-KRas^G12V^ cells (Supplementary Figure S16). Taken together, these results depict the capability of the present methodology to capture and quantify relative changes in protein regulation within the Ras pathway and capture the extent of the biological changes secondary to constitutive activation of the oncogenic KRas^G12V^. These results validate the utility of the selected cell line model.

### SGM proteomics allows for direct development of MS-based assays employing heavy-labeled peptide standards for quantitation of differentially expressed cell-surface proteins

Capitalizing on the off-line SCX-based peptide fractionation to provide for sensitive measurements and enhanced dynamic range of LC-MS analysis [19], we sought to examine the possibility of developing an antibody-free assay for direct MS-based quantitation of identified cell-surface proteins using synthetic heavy peptide standards using parallel reaction monitoring (PRM) [89]. PRM has been successfully used for multiplex quantitation of cytosolic [90] or soluble serum proteins [91]. Towards that goal, we selected xenotropic and polytropic retrovirus receptor 1 (XPR1) as a model surface protein that was found upregulated on the MCF10A-KRas^G12V^ surface via SGM proteomics (Supplementary Table S7B). ThePRM-basedproof-of-principle experiment was carried out on the same instrument used for SGM LC-MS analysis by utilizing the synthetic heavy-labeled peptide standard selected from the list of peptides already identified in the discovery phase via SGM proteomics (Supplementary Figure 17A). The results of the developed XPR1 assay (Supplementary Figure S17B–S17C) were concordant with WB-based cross-validation, depicting the upregulation of XPR1 on the surface of MCF10A-KRas^G12V^ cells. These results demonstrate the utility of the present approach for the development of antibody-free PRM-based quantitative assays for direct quantitation of surface/membrane proteins, similar to the previously described strategy targeting soluble cytosolic and/or serum protein species [90, 91]. The results of the proof-of-principle experiment also validate the utility of the off-line SCX-based peptide fractionation for direct antibody-free multiplex quantitation of cell-surface proteins using heavy-labeled internal peptide standards.

## DISCUSSION

MS-based proteomics has been increasingly used in cell-surface marker/target discovery due to the limitation of genomics to provide explicit information about the status of post-translational modifications and/or subcellular location of a given target [92, 93]. In spite of the importance of KRas mutants in cancer biology, there are no reports to date on in-depth proteomic profiling of the surface of cancer cells expressing KRas mutants. There have been a few proteomic studies targeting whole-cell lysate of cancer cells expressing oncogenic Ras [94, 95].

The present investigation begins to address the basic shortage of viable targets on the surface of cancer cells expressing KRas mutants. Using combined targeted CSC technology and global SGM proteomics, a non-redundant cell-surface map/catalogue of 504 differentially regulated proteins identified on the surface of the MCF10A-KRas^G12V^ cells was generated using MCF10A-EV as a control. This map provides detailed qualitative and broad quantitative information on proteins accessible at the KRas^G12V^ surface, enabling reasonable selection and ranking of putative targets based on their detectability and relative abundance estimated via spectral counting–based quantitation.

In addition, this investigation revealed enrichment/activation of functional protein networks regulating cell motility, cellular protrusions formation, proliferation, cellular assembly, drug metabolism, and embryonic development. Activation of these functional networks is consistent with the KRas-driven malignant transformation involving activation of signaling cascades implicated in cell migration, invasion, EMT, and KRas and cancer stem cells regulation [29].

Among cross-validated surface proteins, more than a few of the identified targets were implicated in KRas-driven tumorigenesis and were previously investigated by others employing common molecular biology techniques and proposed as surface targets, such as CD147, CDCP1, IGF1R, PTPRJ, and TGFBR2. The rediscovery and/or confirmation of known drug targets using MS-based proteomics is indicative of the utility of the present methodology for confident identification of novel targets. For the remaining cross-validated surface targets, which include ABCA2, ANTXR1, CDH4, CELSR1, PROCR, VANGL1, SLC9A7, and XPR1, the literature search showed no explicit connection to the oncogenic KRas signaling. Hence, these surface molecules may serve as the first line of prospective targets to be further investigated using cancer cell lines expressing oncogenic KRas mutants or validated in human KRas-driven cancers using tissue protein arrays.

For the first time, this study revealed co-localization of CD147 and CDCP1 along enriched actin filaments on the tip of cell-surface protrusions (Figure 6). This allowed us to hypothesize that concomitant use of therapeutic antibodies against gene products of CD147 and CDCP1, combined with actin polymerization inhibitors (e.g., formin), may facilitate a more effective arrest of tumor invasion and metastasis. This is important since, in spite of successful initial treatment via surgery alone or in combination with radiation and chemotherapy, over 90% of deaths from KRas-driven cancers are caused by metastatic disease facilitated by invadopodia-driven migration/invasion of cancer cells. Hence, the CD147-CDCP1-actin co-localization warrants further investigation using standard molecular biology techniques (e.g., immunoprecipitation) and/or RNAi pharmacological interference, which is beyond the scope of this study. While others have demonstrated the relationship between Ras and CD147 using the same model [96], this analysis revealed for the first time the upregulation of CD147 not only on the surface of the MCF10A-KRas^G12V^model cell line but also on the surface of pancreatic, lung, and colon cancer cell lines expressing KRas mutants at the endogenous levels. These findings make CD147 worthy of further investigation as a potential universal KRas surface marker/target, regardless of the origin of the primary tumor.

In addition to wide-range profiling of the MCF10A-KRas^G12V^ surface, the use of two orthogonal proteomic platforms (CSC technology and SGM proteomics) allowed for in-depth bioinformatic processing of raw MS-data, resulting in extensive coverage (70%) of the Ras pathway per se beyond the outer leaf of the cell membrane. Besides the expected activation of the ERK/MAPK signaling, it revealed the activation of pancreatic adenocarcinoma signaling, non-small cell lung cancer signaling, colorectal cancer metastasis signaling, actin nucleation by ARP-WASP complex signaling, and other canonical pathways depicted in Supplementary Table S9. In light of increasing evidence supporting a role of axon guidance genes in cell migration, invasiveness, survival, metastasis, and angiogenesis in various cancers (including pancreatic cancer), [97, 98] it is important to point out the similarity of our proteomic findings with genomic results revealing axonal guidance signaling as the most deregulated on a large cohort of pancreatic (stage I and II) ductal adenocarcinomas [85]. Notably, this approach also allowed the ability to identify/quantify all Ras isoforms in isoform-specific manner, exemplified by the concordance of our results with the study reported by the Bar-Sagi group [86] employing standard molecular biology techniques reporting for the first time, as well as the dependence of KRas mutants on wild-type HRas and NRas isoforms. Taken together, these results depict the capability of the present methodology to capture and quantify relative changes in protein regulation within the Ras pathway and capture the extent of biological changes secondary to constitutive activation of the oncogenic KRas^G12V^. These results validate the utility of the selected cell line model.

While a plethora of target and/or biomarker candidates have been discovered and proposed over the last decade using genomic and/or MS-based profiling, only a few of them have been implemented in the clinic so far. High-quality immunoassays (e.g., multiplex ELISA, multiplex MS-based immuno-MRM) still rely on the availability of specific antibodies against intact proteins and/or peptides, available only for a small subset of the human and/or mouse proteins [99, 100]. Hence, the principal obstacle for translation of putative targets from discovery to the validation phase using large clinical sample cohorts is the lack of high-throughput assays for their reproducible and multiplex measurements.

To investigate the suitability of the described proteomic platform for direct development of antibody-free PRM-based quantitative assays for selected surface proteins identified via SGM proteomics, we selected xenotropic and polytropic retrovirus receptor 1 (XPR1) as a model molecule that has been previously characterized as a genuine cell surface protein [101]. The assay was successfully developed and validated. The present SGM platform offers multiple advantages for MS-based quantitation of cell-surface proteins. First, the discovery step and the quantitation step are performed using the same sample and the same LC-MS instrument/platform. Second, the selection of internal peptide standards is driven by experimental data and does not require development/use of complex prediction algorithms [102]. Third, this is an antibody-free MS technique amenable to multiplex quantitative measurements via high speed and high resolution/accuracy LC-MS [91, 103]. While the involvement of XPR1 in the biology of KRas-driven cancers has not been previously reported, it is plausible to hypothesize that its downstream upregulation may be involved in the regulation of cell proliferation [104, 105].

At first sight, choosing the KRas^G12V^ transfected MCF10A cell line may appear to be a drawback, since it may not closely resemble the biology of cell lines expressing KRas mutants endogenously. However, there are certain advantages to using it. First, the fidelity of any cell-surface map depends heavily on the choice/quality of the selected “normal” control cell line. Since a majority of currently available “normal” epithelial cell lines are genetically manipulated, there is still a lot of debate among molecular biologist about how accurately the biology of these “normal” cell lines (e.g., BEAS-2B, CRL-4307), immortalized via aggressive genomic manipulations (e.g., viral vectors, hTERT), resemble biologically normal cells. Second, the MCF10A cell line is the first naturally immortalized human epithelial cell line that arose spontaneously from a mortal non-malignant mammary epithelium without any genomic manipulations [106]. Third, MCF10A cells retain a normal diploid chromosome pattern in cell culture and express normal p53 [107]. Fourth, the parental breast MCF10A cell line does not grow or produce tumors in immunocompromised nude mice [108]. However, subcutaneously injected MCF10A-KRas^G12V^ cells form tumors in immunodeficient BALB/c female nude mice [109]. Fifth, the use of the transformed mammary MCF10A-KRas^G12V^ cell line alleviates the organ/tumor-specific bias (e.g., pancreas, lung, colon). Therefore, the effects of KRas^G12V^ signaling observed in the context of the mammary cell line may be interpreted as a general response, since KRas mutants play a negligible role in the biology of breast cancer [110]. Furthermore, the upregulation of CD147 on the surface of the three KRas cell lines (KP3, H2444, and SW620) expressing CD147 endogenously, as shown by IF analysis, supports this hypothesis as well as the generalization of this data to other types of cancers and/or cancer cell lines.

Another limitation of this study is of a general nature. A tumor is not only about cancer cells (e.g., parenchyma) but is also about the tumor microenvironment (e.g., stroma), which contains immune and non-immune tumor cells. Any malignant tumor larger than 2 mm in diameter cannot survive without stroma (e.g., blood vessels) providing the nutrients necessary for its survival [111]. It is certainly difficult to predict how much of the “Petri dish biology,” devoid of a natural tumor microenvironment, is reflective of the actual cancer biology taking place in KRas-driven tumors in tissues. Therefore, innovative proteomic approaches capable of efficient cell-surface protein profiling in tissue are sorely needed.

In summary, this investigation provides an in-depth reference map of the KRas^G12V^ surface that may facilitate more rapid selection and further validation of candidate targets for the generation of therapeutic antibodies against surface proteins in the context of immune therapy for KRas-driven cancers. The large-scale coverage is achieved by combining the CSC technology, which targets specifically N-glycosylated cell-surface protein species, with global SGM proteomics capable of identifying cell-surface proteins regardless of their post-translational modification status.

Using multistep biology-gnostic bioinformatic filtering (IPA^®^), combined with biology-agnostic statistical processing and manual validation, we were able to identify, select, prioritize, and cross-validate a subset of novel and known KRas surface targets. In addition to cell-surface targets selected/validated in this study, there exists a multitude of interesting gene products contained in the generated surface map that may represent a starting point for the characterization and development of novel targets. Also, this map may facilitate a better understanding of the biology of cancers driven by KRas mutants and may serve as the resource for prospective studies and development of antibody-free, high-throughput MS-based quantitation assays for cell-surface targets.

## MATERIALS AND METHODS

### Materials

Biocytin hydrazide was purchased from Biotium (Hayward, CA). Streptavidin Plus UltraLink Resin was purchased from Pierce (Rockford, IL). RapiGest SF surfactant was obtained from Waters (Milford, MA). Tris 2-carboxyethylphosphine, Bond Breaker™ (TCEP) and iodoacetamide (IAA) were obtained from Pierce (Rockford, IL). Sequence-grade modified trypsin was obtained from Promega (Madison, WI). PNGase F was obtained from New England BioLabs (Ipswich, MA). The antibodies used for western blot and immunofluorescence analyses were from the following sources: anti-β-actin, anti-H-Ras (C-20), anti-N-Ras (F155), and anti-KRas (F234) were from Santa Cruz Biotechnology (Dallas, TX); anti-ANTXR1, anti-BSG, anti-IGF1R, anti-PTPRJ, anti-SOS2, anti-CELSR1, anti-SLC9A7, and anti-TGFBR2 were from OriGene Technologies (Rockville, MD); anti-CDCP1 was from Thermo Fisher Scientific (Waltham, MA); anti-CDH4 was from Sigma-Aldrich (St. Louis, MO); anti-PROCR was from LSBio (Seattle WA); anti-ABCA2, anti MCAM (CD146), and anti-XPR1 were from Abcam (Cambridge, United Kingdom); and anti-VANGL1 was from R&D Systems (Minneapolis, MN). All other chemicals were purchased from Sigma-Aldrich (St. Louis, MO). Heavy XPR1 peptide standard H_2_N-INQLISETEAVVTNELEDGDR^-OH, containing (13C)6H14(15N)402 labeled C-terminal arginine [Mass Shift +10], and the corresponding light XPR1 peptide H2N-INQLISETEAVVTNELEDGDR-OH, were from New England Peptide™ (Gardner, MA).

### Cell lines and culture methods

MCF10A-KRas^G12V^ and empty vector transfected MCF10A-EV cells were gifts from James Wells' lab. Both cell lines were cultured in DMEM/F12 (Life Technologies, Grand Island, NY) and supplemented with 5% Horse Serum (Life Technologies), 20 ng/ml EGF (Life Technologies), 0.5 μg/ml Hydrocortisone (Sigma), 100 ng/ml Cholera Toxin (Sigma), and 10 μg/ml Insulin (Sigma).

### Scanning electron microscopy

The samples were fixed in a cocktail of 4% formaldehyde and 2% glutaraldehyde in 0.1M cacodylate buffer and post-fixed using a 1% osmium tetroxide solution. They were then dehydrated in a series of graded alcohols and air dried after a final dehydration course of tetramethylsilane. Subsequently, the samples were sputter coated with a thin layer of iridium and imaged utilizing a Hitachi S-4500 field emission scanning electron microscope (Tokyo, Japan).

### Phenotypical cancer cell assays

The description of Boyden chamber migration, Boyden chamber invasion, and anchorage-independence assays is accessible in Supplementary Information.

### CSC technology: sample preparation, LC-MS and bioinformatic analysis

Samples were prepared as outlined in the targeted CSC proteomics sequence depicted in the experimental workflow (Figure 1). Briefly, equal amounts (approximately 10^8^) of MCF10A-KRAS^G12V^ and MCF10A-EV cells were suspended in labeling buffer and prepared using the previously described protocol. The samples were prepared on two independent occasions (i.e., two biological replicates). The resulting glycopeptide specimens were cleaned using a C18 spin column (Pierce) prior to the LC-MS analysis. Samples from each preparation were injected three times (i.e., three technical replicates) into the high resolution/accuracy hybrid MS.

Hydrazide-captured/enriched glyco-peptides from cell-surface proteins were analyzed using nano-flow reversed phase (RP) LC-MS using the Agilent 1100 nano-flow LC system coupled on-line to an Orbitrap Elite mass spectrometer (ThermoElectron, San Jose, CA). The final peptide mixture, reconstituted in a total of 20 μL of 0.1% TFA, was analyzed in triplicates by injecting 5 μL of the sample on a RP column (75 μm i.d. × 10 cm fused silica capillary with a flame-pulled tip) and slurry-packed in-house with 5 μm, 300 Å pore size C-18 stationary phase (Phenomenex, Torrance, CA). After sample injection, the column was washed for 20 min with 98% mobile phase A (0.1% formic acid in water) at a flow rate of 0.5 μL/min. Peptides were eluted from the column using a linear gradient of 2% mobile phase B (0.1% formic acid in ACN) to 40% solvent B for 100 min at a flow rate of 0.25 μL/min, then to 98% B for an additional 20 min. The instrument was operated in a data-dependent mode, using the peptide ion mass to charge range of 400−1800, monitored at the resolution level of 60,000 at m/z 400. Each MS^1^ scan was followed by 16 MS^2^ scans, wherein the 16 most abundant precursor ions were dynamically selected for collision-induced dissociation using normalized collision energy of 35%.

Proteins were identified applying the SEQUEST algorithm-based search against the non-redundant human proteome database (SwisProt release v57.15) utilizing the Proteome Discoverer 1.4 (Thermo). The database search thresholds included: for the monoisotopic peptide precursor ions (MS^1^ spectra), mass tolerance was set at 5 ppm, and for the fragment ions (data-dependent MS^2^ spectra), mass tolerance was set at 0.6 Da. Dynamic amino acid modifications were added for the detection of the following: +0.984 Da for asparagine deamidation (i.e., deamidation of N-glycosylated asparagines via PNGase F treatment), +57.021 Da for cysteine carboxyamidomethylation (i.e., alkylation), and +15.994 Da methionine oxidation. The search allowed for peptides with one tryptic terminus (K, R), allowing for up to two missed cleavage sites. Next, search results were filtered and manually inspected for the presence of peptides containing deamidated asparagine in the context of the N-glycosylation sequence motif (i.e., NxST) to further decrease peptide/protein false discovery rate (FDR). Glyco-proteins identified by a single peptide spectrum match (PSM) were not included in the final dataset.

Spectral counting quantitation of changes in protein regulation between KRas and the EV surface was carried out using PSMs readouts computed by the Percolator algorithm within the Proteome Discoverer software. Percolator relies on semi-supervised machine learning to improve the discrimination between correct and incorrect spectra identifications, taking into account p-value, q-value, and posterior error probability for each peptide match at the selected strict FDR of ≤ 0.01. Significantly upregulated surface proteins were revealed using binomial probability and false discovery rate (FDR) calculations [112].

Protein grouping was employed to increase the quality and reliability of protein identifications and enforce economy in the number of identified proteins. Cell-surface proteins were characterized in accordance with their annotations in the human cell surface protein atlas (CSPA). Selection and prioritization of cell-surface proteins for antibody-based cross-validation using IF and WB analyses were facilitated using PANTHER and IPA^®^ bioinformatic tools.

### SGM proteomics: sample preparation, LC-MS and bioinformatics analysis

Pellets containing approximately 10^7^ cells from MCF10A-KRAS^G12V^ and MCF10A-EV were suspended in buffer containing 25 mM NH_4_HCO_3_ and 1 mM PMSF and lysed by sonication (five 10-second bursts applying 20% intensity) using a Bronson microprobe sonicator. The homogenate was centrifuged at 2000 × g for 10 min to remove cellular debris. The supernatant was ultra-centrifuged (37,000 rpm) for 1.5 hrs at 4°C using a Beckman MLS50 rotor (Brea, CA). The supernatant was discarded, and the crude membrane fraction was subjected to a modified carbonate treatment as previously described [19, 113]. The resulting membrane pellets from MCF10A-KRAS^G12V^ and MCF10A-EV cells were re-suspended in 50 mM NH_4_HCO_3_ containing 1 mM PMSF, and the protein concentration was determined using the BCA Protein Assay Kit (Pierce). Next, the proteins were reduced by 3 mM TCEP for 30 min, followed by alkylation using 5 mM IAA for 30 min at 37°C. Two equal protein aliquots, 500 μg each, were lyophilized and then solubilized in 500 μL of buffer containing 60% (v/v) CH_3_OH and 0.1% acid-cleavable surfactant in 25 mM NH_4_HCO_3_. Membrane proteins were digested using trypsin as previously described. After digestion, samples were lyophilized and dissolved in 200 μL of 45% acetonitrile containing 0.1% formic acid and fractionated using SCX chromatography as previously described. The 96 SCX fractions were pooled into 12 fractions based on the peptide chromatography profile, lyophilized to dryness, and reconstituted in 0.1% TFA. SCX peptide fractions were analyzed using nano-flow reversed phase (RP) LC-MS using an Agilent 1100 nano-flow LC system coupled on-line to an Orbitrap Elite mass spectrometer (ThermoElectron, San Jose, CA). Each SCX fraction was reconstituted in approximately 20 μL of 0.1% TFA and analyzed in duplicate by injecting 5 μl (approximately 0.2 μg/μl peptide concentration) as described above.

Proteins were identified by applying the SEQUEST algorithm-based search against the non-redundant human proteome database (SwisProt release v57.15) utilizing the Proteome Discoverer 1.4 (Thermo). Proteins identified by a single peptide matching spectrum (PMS) were not included in the final dataset. Protein grouping was employed to increase the quality and reliability of protein identifications and enforce economy in the number of identified proteins. The database search thresholds included: for the monoisotopic peptide precursor ions (MS^1^ spectra), mass tolerance was set at 5 ppm; and for the fragment ions (data-dependent MS^2^ spectra), mass tolerance was set at 0.6 Da. Dynamic amino acid modifications were added for the detection of the following: +57.021 Da for cysteines (i.e., carboxyamidomethylation), and +15.994 Da for methionines (i.e., oxidation), at least one tryptic terminus (K, R), and up to two missed peptide cleavages. A strict peptide false discovery rate of ≤ 0.01 was set using Percolator-based statistical evaluation relying on p-value, q-value, and posterior error probability for each match. Peptide/protein quantification and statistical assessment of significance for the SGM dataset was calculated in the same manner as the above described processing of the CSC dataset.

Search results were first analyzed using PSORT and TMHMM algorithms to characterize membrane proteins. The IPA^®^ knowledge database filtering was used next to map genuine cell-surface proteins contained within the membrane protein dataset. Finally, to uncover N-glycosylated proteins within the cell surface proteome mapped via SGM proteomics, the IPA^®^ knowledge database generated subset was run against the human cell surface protein atlas (CSPA) annotations. Surface targets selection/prioritization for antibody-based cross-validation using IF and WB analyses relied primarily on bioinformatic filtering using PSORT, TMHMM, PANTHER, IPA^®^, and statistics.

### Generation of non-redundant MCF10A-KRAS^G12V^ cell-surface map

Subtractive proteomic analysis was used to reveal a non-redundant list of targets identified solely on the surface of MCF10A-KRas^G12V^cells via both CSC and SGM proteomics. Comparative proteomic analysis that relies on spectral counting to quantify relative changes in protein abundances was used to reveal and generate the non-redundant list of proteins upregulated at the surface of MCF10A-KRas^G12V^cells.

### Quantitative XPR1 assay development using PRM

#### PRM assay LC-MS analysis

A total of 6 μL of membrane digest from KRas-transfected and EV-transfected MCF10A cells was mixed with 6 μL of XPR1 heavy peptide (1ng/μL). A total of 2 μL of mixture was analyzed by liquid chromatography-mass spectrometry (LC-MS) analysis using an Agilent 1100 nano-flow LC system coupled on-line with an Orbitrap Elite instrument (Thermo Electron, San Jose, CA). RP columns (75 μm i.d. × 10 cm fused silica capillary with a flame-pulled tip) were slurry-packed in-house with 5 μm, 300 Å pore size C-18 stationary phase (Phenomenex, Torrance, CA). After sample injection, the column was washed for 20 min with 98% mobile phase A (0.1% formic acid in water) at a flow rate of 0.5 μL/min. Peptides were eluted from the column using a linear gradient of 2% mobile phase B (0.1% formic acid in ACN) to 42% solvent B for 40 min at a flow rate of 0.25 μL/min, then to 98% B for an additional 10 min.

#### Parallel reaction monitoring on LTQ Orbitrap Elite

PRM was performed as described previously [89] with the following alterations due to differing instrument architecture. Reverse phase nano-flow eluted ions were ionized in positive mode with a voltage of 1.5kV and an ion transfer tube temperature of 200°C. Two fragment windows were alternatively selected for fragmentation for each run to obtain MS/MS fragments for quantification. The first window was centered on 782.4 and the second on 785.6. Ions were isolated in the linear ion trap with a symmetrical 1.5 Da isolation window, and automatic gain control was utilized to obtain a target of 1e5 charges, or a maximum of 300 ms if the target could not be achieved, as set in the master tune file. The isolated ions were fragmented with CAD fragmentation in the ion trap, and the resulting daughter ions were transferred for high-resolution accurate mass analysis in the Orbitrap with a resolution of 17,500 at 400 m/z.

#### Data processing

LC-MS data files were loaded into Pinpoint version 1.4.0 (Thermo Fisher Scientific). An *in silico* digest of the intact XPR1 protein from SwissProt was digested with standard tryptic parameters, allowing up to two missed cleavages. A second version of the intact protein was loaded with the same parameters, with heavy labeling of the K and R sets as static modification on every amino acid, and it was used as the internal control for quantification. Fragment ions were quantified for the five most intense of the predicted transitions that met a mass cutoff of 3 ppm. The peak intensity was normalized against the heavy internal standard by the default parameters in Pinpoint. Extracted ion chromatograms were manually obtained in Xcalibur Qual Browser (Thermo Fisher Scientific) to manually validate the quantification values obtained by Pinpoint.

### WB analysis

Cells were lysed in 25 mM of NH4HCO3 buffer supplemented with 1 mM PMSF and homogenized by five cycles of 10-second sonication (20% intensity) using Bronson microprobe sonicator. The homogenate was centrifuged at 1000 × g for 10 min to remove unbroken cells and cellular debris. The supernatant was ultracentrifuged at 37,000 rpm for 1.5 hrs using a Beckman MLS50 rotor (Brea, CA). The membrane pellet was resolubilized in 25 mM of NH4HCO3 buffer, and the protein concentration of the solution was determined with the BCA Protein Assay Kit (Pierce). An equal amount of protein was run on SDS-PAGE (Life Technologies). Resolved proteins were transferred onto a nitrocellulose membrane (Bio-Rad, Hercules, CA), blocked with 5% non-fat dried milk in PBST (PBS with 0.05% Tween), incubated with primary Ab at 4°C overnight, washed with PBST, and probed with HRP-conjugated secondary Ab (Johnson ImmunoResearch, West Grove, PA). Immunoreactive bands were visualized by colorimetric detection using the Opti-4CN Substrate Kit (Bio-Rad). Expression of each target was quantitated using Image J and normalized to respective ACTB level.

### Immunofluorescence analysis

Cells were cultured on coverslips and washed with cold PBS three times, fixed in 4% formaldehyde, permeabilized with 0.1% Triton X-100, and blocked with Odyssey™ Blocking Buffer (Li-Cor, Cambridge, UK) for 1 hr. Cells were then incubated with primary antibodies overnight at 4°C, followed by incubating with secondary antibodies conjugated with Alexa Fluor 488 (Invitrogen) for 2 hrs. Cells were also stained with 4,6-diamidino-2-phenylindole (DAPI) (Invitrogen) to visualize the nuclei.

### Fluorescent microscopy

Wide-field images were acquired on a Nikon Eclipse Ti inverted microscope, using a 60x NA 1.42 Plan Apo objective. The microscope was equipped with a 64 μm pixel CoolSNAP HQ^2^ camera (Photometrics) and Intensilight C-HGFIE illuminator. 200 nm Z-sections were acquired. ImageJ (National Institute of Health, Bethesda, MD) software was used to make maximum intensity projections and to assemble figures.

Structured illumination microscopy (SIM) that relies on a grid pattern to provide higher resolution images was performed on N-SIM, Nikon Inc., equipped with an Apo TIRF 100x NA 1.49 Plan Apo oil objective, 405, 488, 561, and 640 nm excitation lasers, and back-illuminated 16 μm pixel EMCCD camera (Andor, DU897). 100 nm Z Sections were acquired in 3D SIM mode, generating 15 images per plane. Channels were corrected for chromatic shift based on the signals of 100 nm multi-spectral fluorescent spheres (TetraSpeck beads, Invitrogen) that were included in the mounting medium. For 3D visualization, we used the NIS-elements software package. To allow comparison of signal intensities, cells were imaged using identical imaging settings, and images were processed identically during figure assembly.

## SUPPLEMENTARY FIGURES, TABLES AND METHODS




