# Supplementary material for: Comparative proteomics of a model MCF10A-KRasG12V cell line reveals a distinct molecular signature of the KRasG12V cell surface
Source: Oncotarget. 2016 Nov 24;7(52):86948–71. doi: 10.18632/oncotarget.13566 (PMC5341332; doi:10.18632/oncotarget.13566)
Supplement: Supplementary file 1 [file oncotarget-07-86948-s001.pdf]

Comparative proteomics of a model MCF10A-KRas^{G12V} cell line reveals a distinct molecular signature of the KRas^{G12V} cell surface

SUPPLEMENTARY FIGURES, TABLES AND METHODS

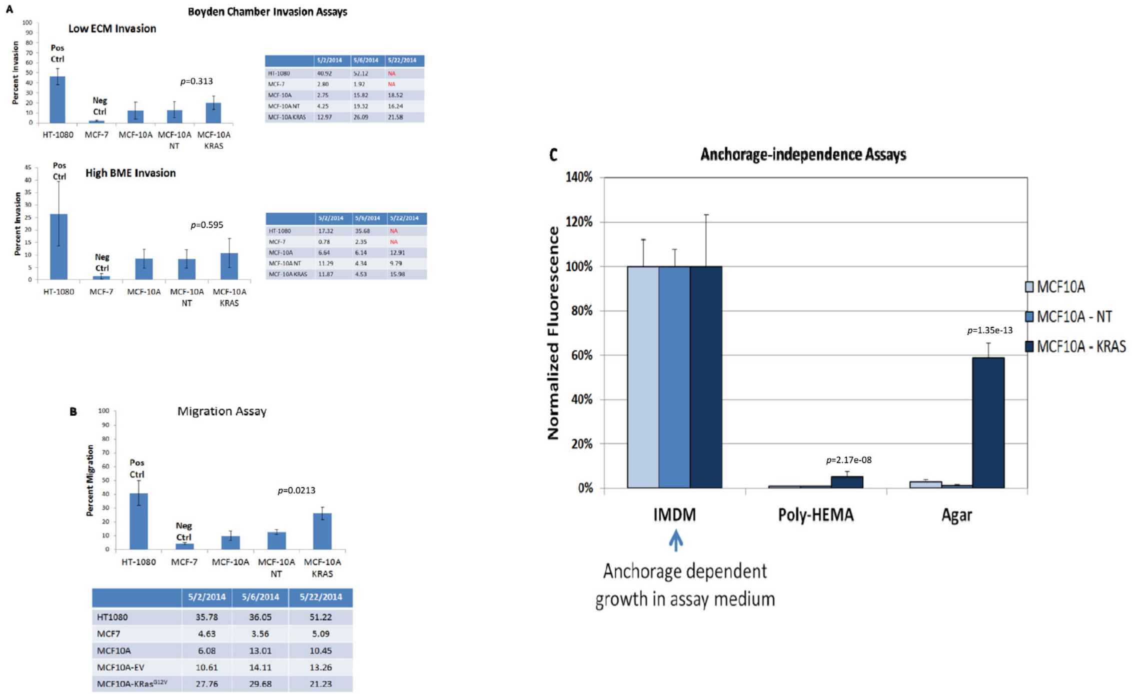

Supplementary Figure S1: Phenotypic cancer cell assays. A. Low ECM and high BME invasion assays. **B.** Migration assay. **C.** Anchorage-independence assays, showing measurements obtained on three different occasions, consistent with malignantly transformed EMT-like phenotype of MCF10A-KRas^{G12V} cells. For Figures (A) and (B), the p-values were obtained from a two-sample t-test comparing MCF10A-NT(EV) against MCF10A-KRAS. For Figure (C), the p-values were obtained from a chi-square test of the normalized values from the anchorage-independent assays. Since this test will not work for small values, the poly-HEMA were increased by a factor of ten to make them comparable in magnitude to the Agar values.

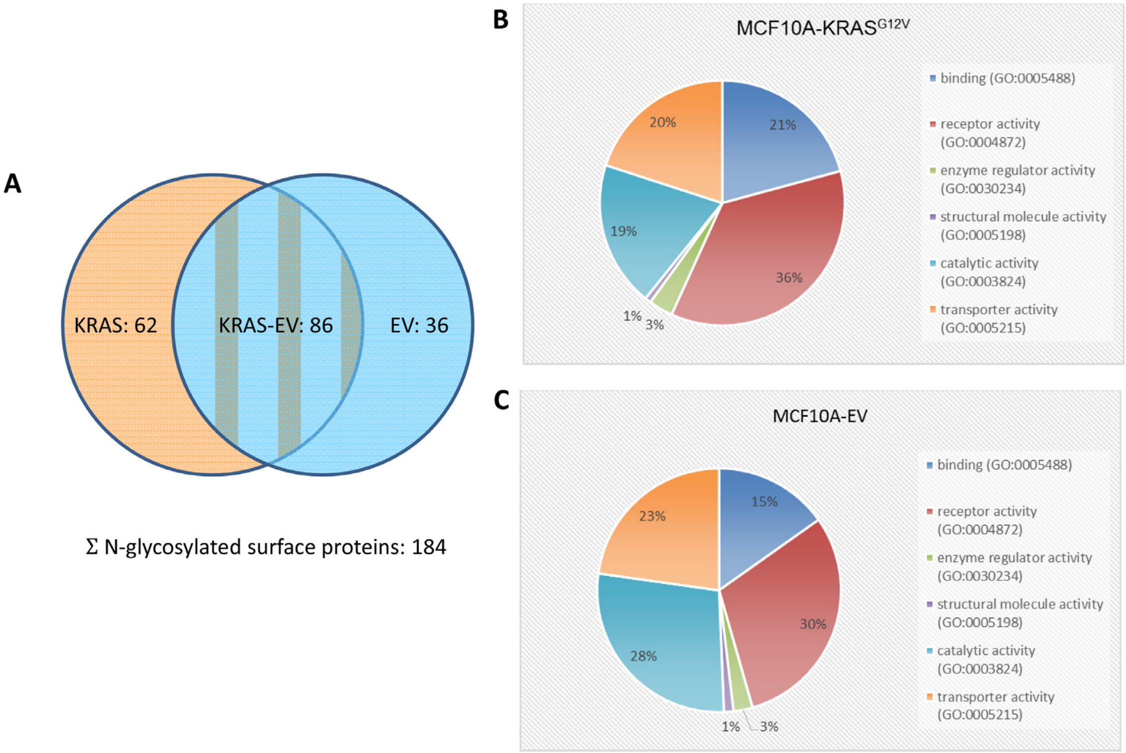

Supplementary Figure S2: Venn diagrams showing overlapping glycoprotein identifications on the surface of MCF10A-KRas^{G12V} and MCF10A-EV cell lines using CSC proteomics A. Distribution of PANTHER molecular functions of identified surface proteins in MCF10A-KRas^{G12V} **B.** and MCF10A-EV **C.** using CSC proteomics.

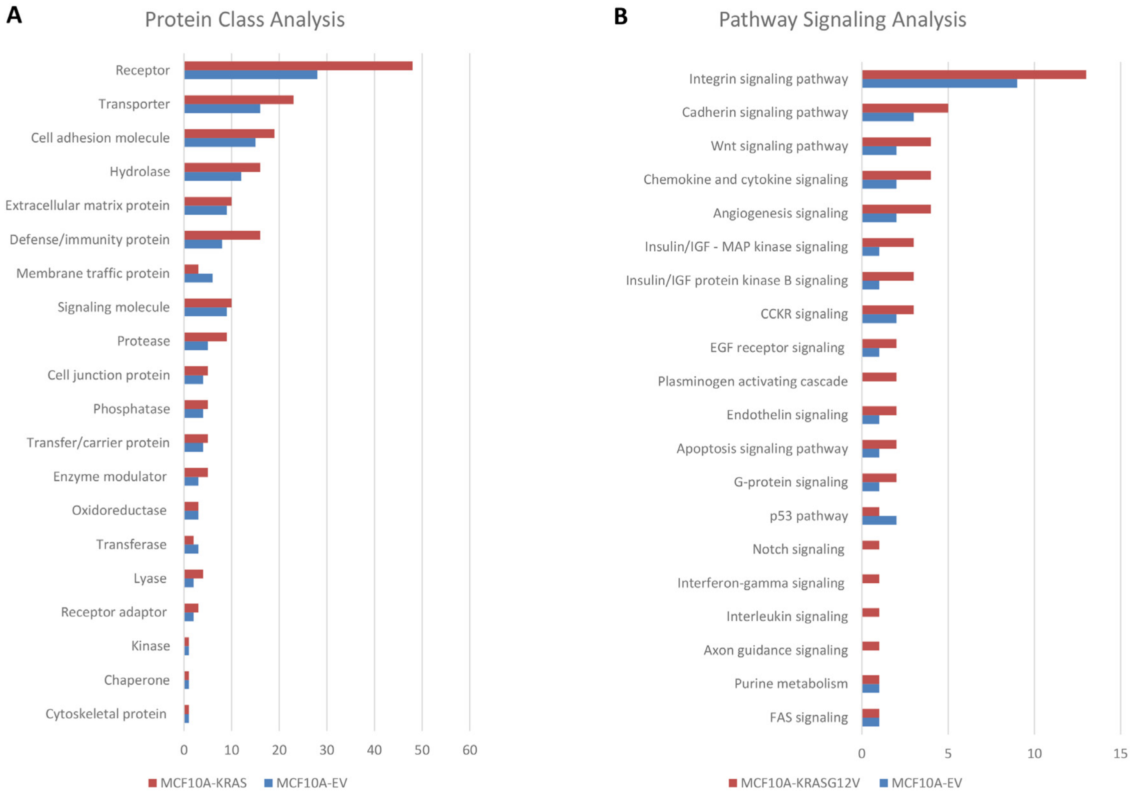

Supplementary Figure S3: PANTHER protein class analysis A. and signaling pathway analysis **B.** of a complement of glycoproteins mapped on the surface of MCF10A-KRas^{G12V} and MCF10A-EV cells using CSC technology.

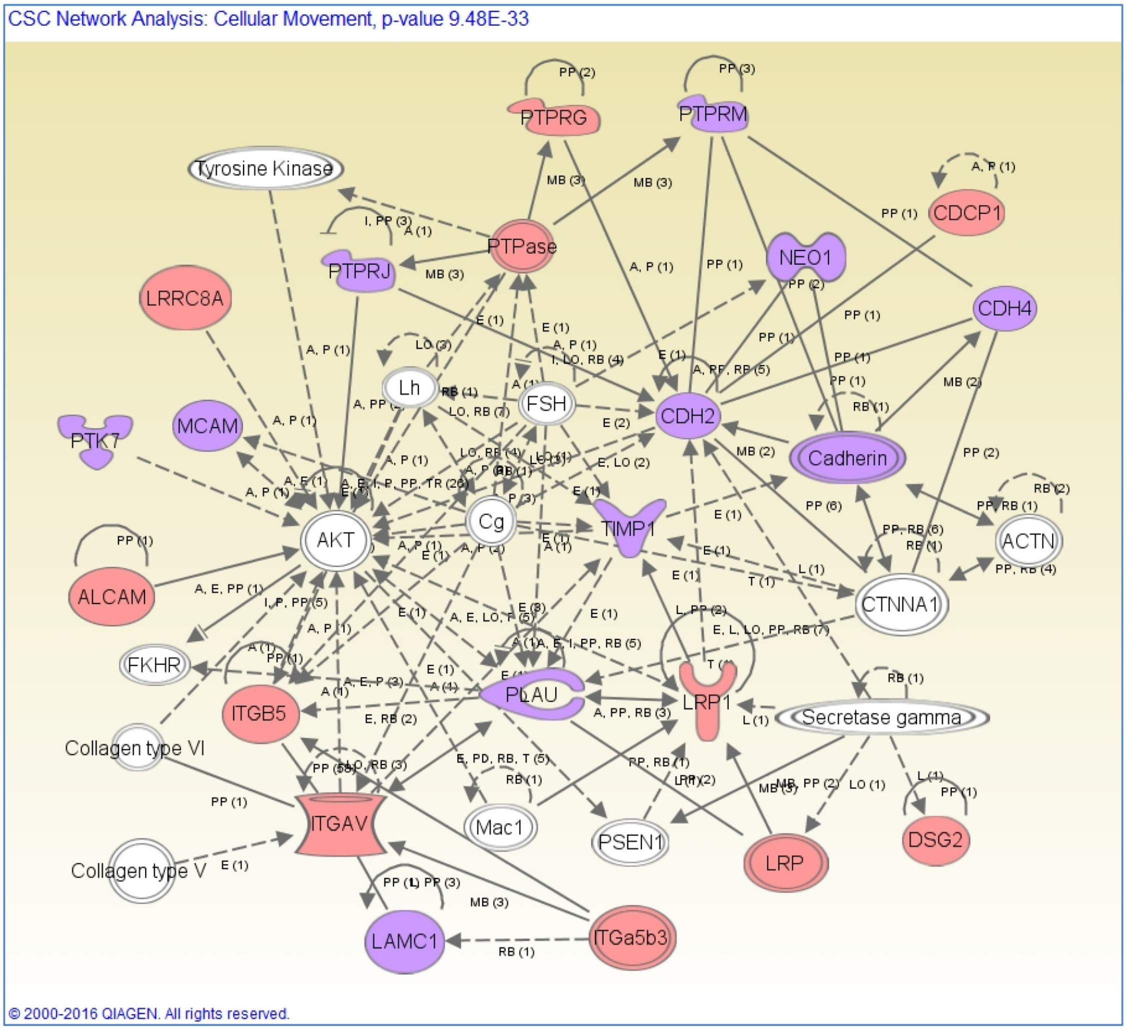

Supplementary Figure S4: IPA ® Network Analysis: Cellular Movement, p-value 9.48E-33. Purple color depicts proteins unique to the $KRas^{G12V}$ surface, red color depicts proteins up-regulated on the $KRas^{G12V}$ surface. Solid lines show direct interactions, while dotted lines show indirect protein-protein interactions described in the literature.

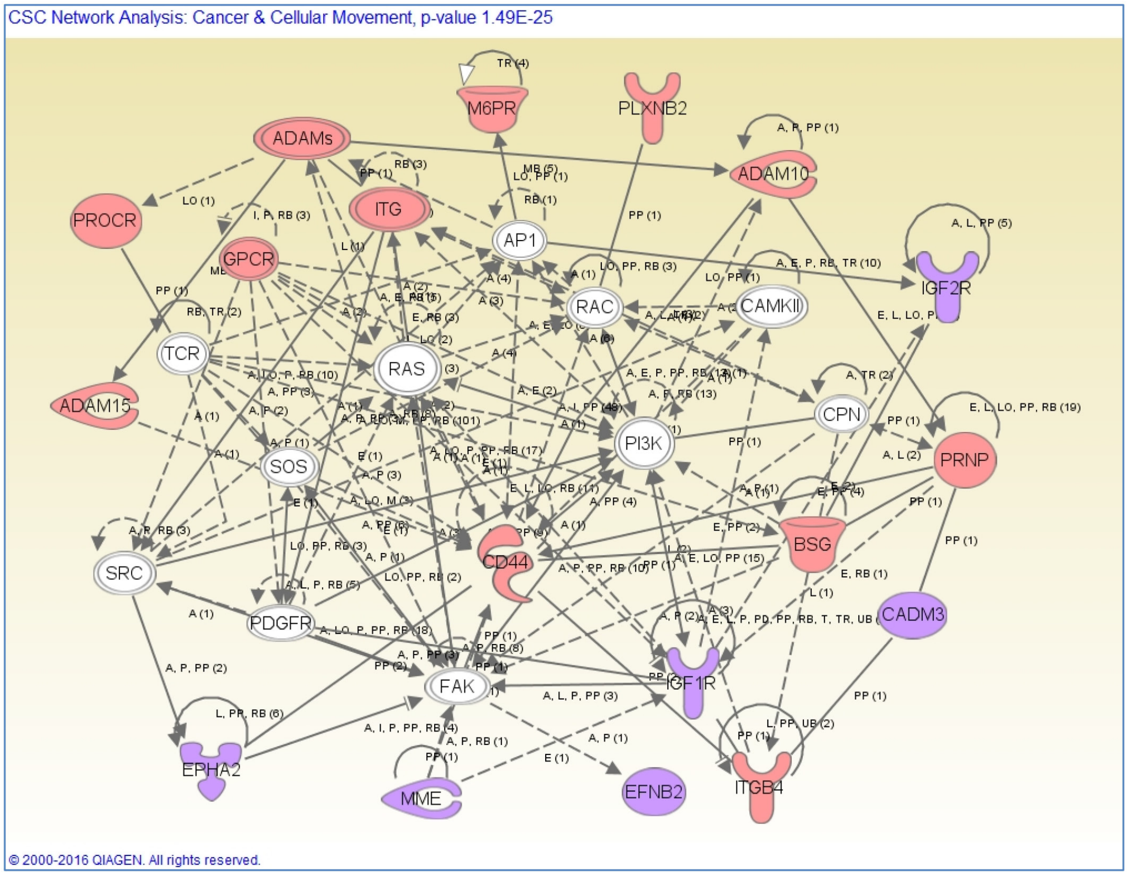

Supplementary Figure S5: IPA ® Network Analysis: Cancer & Cellular Movement, p-value 1.49E-25. Purple color depicts proteins unique to the KRAS^{G12V} surface, red color depicts proteins up-regulated on the KRas^{G12V} surface. Solid lines show direct interactions, while dotted lines show indirect protein-protein interactions described in the literature.

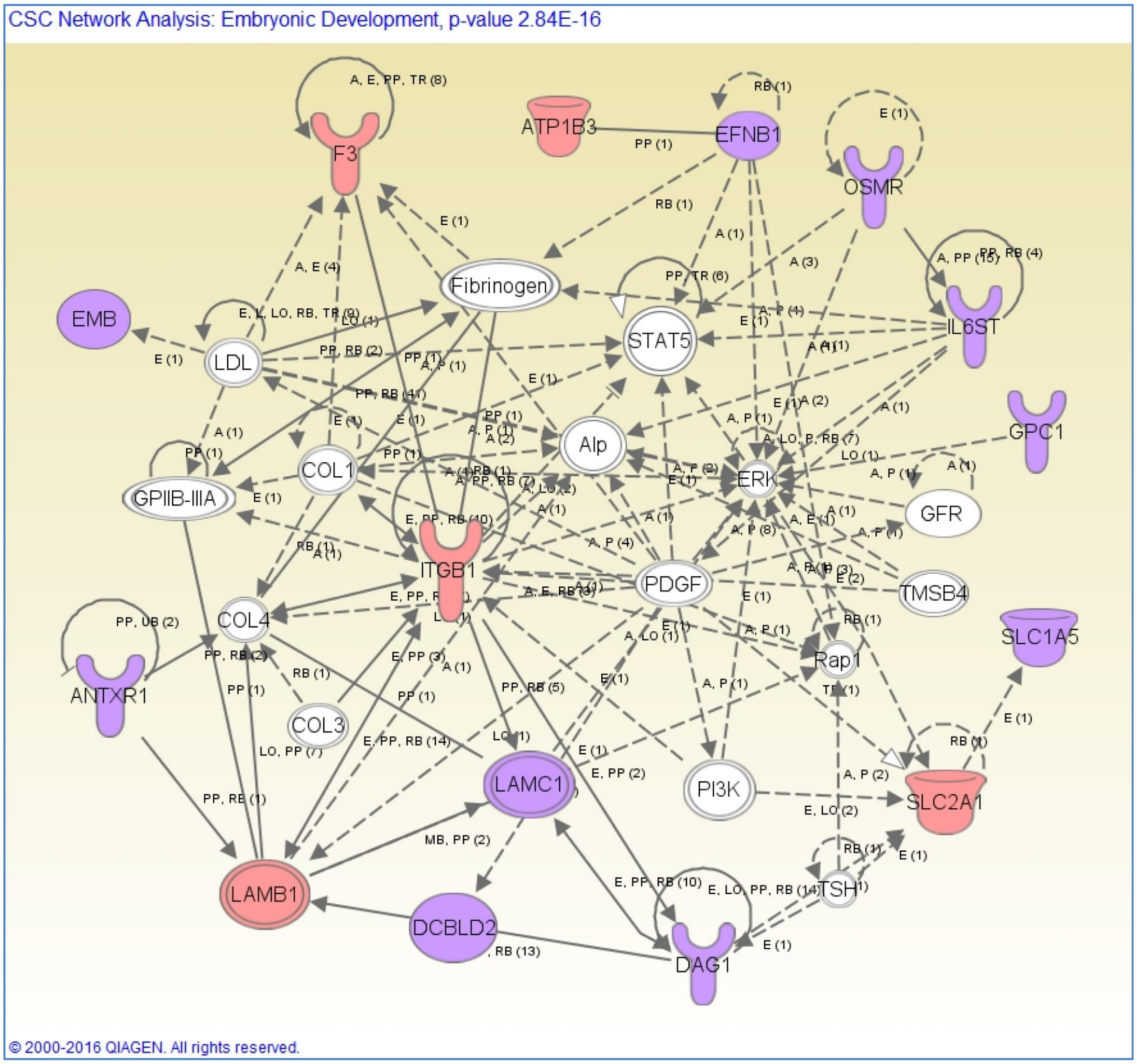

Supplementary Figure S6: IPA ® Network Analysis: Embryonic Development, p-value 2.84E-16. Purple color depicts proteins unique to the KRas^{G12V} surface, red color depicts proteins up-regulated on the KRas^{G12V} surface. Solid lines show direct interactions, while dotted lines show indirect protein-protein interactions described in the literature.

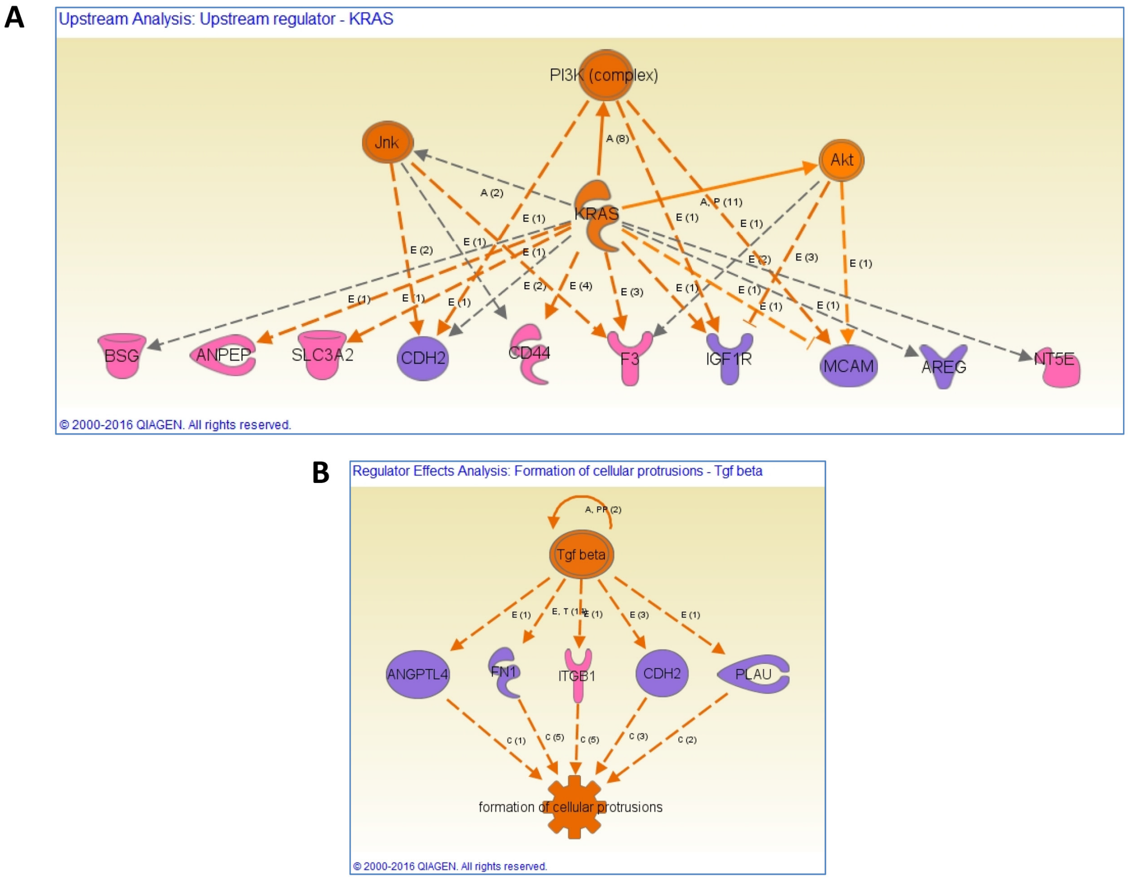

Supplementary Figure S7: A. IPA® Upstream Analysis revealed KRas as an activated upstream regulator, z-activation score 1.823, p-value 1.76E-06. **B.** IPA® Regulator Effects Analysis revealed ANGPTL4, FN1, ITGB1, CDH2, and PLAU as downstream targets of TGf beta in the context of formation of cellular protrusions/invadopodia. Purple color depicts proteins unique to the $KRas^{G12V}$ surface, red color depicts proteins up-regulated on the $KRas^{G12V}$ surface. Solid lines show direct interactions, while dotted lines show indirect protein-protein interactions described in the literature.

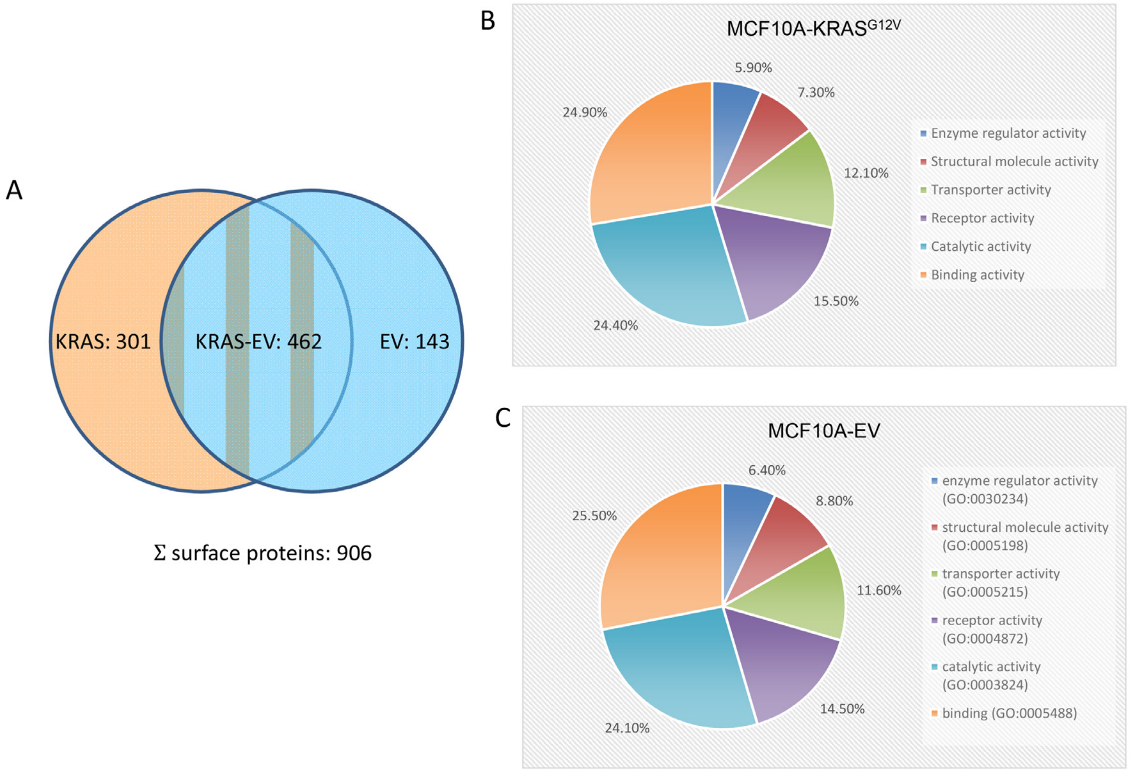

Supplementary Figure S8: A. Venn diagrams showing overlapping protein identifications on the surface of MCF10A-KRas^{G12V} and MCF10A-EV cell lines using SGM proteomics. Distribution of PANTHER molecular functions of identified surface proteins in MCF10A-KRas^{G12V} **B.** and MCF10A-EV **C.** using SGM proteomics.

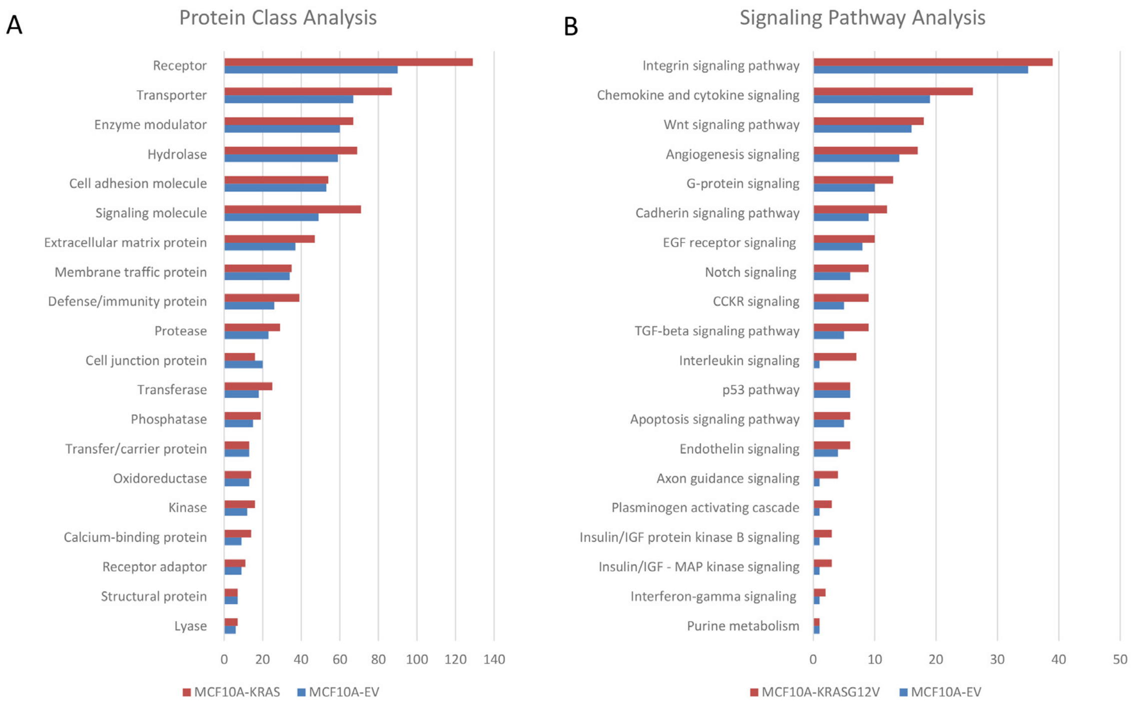

Supplementary Figure S9: PANTHER protein class analysis A. and signaling pathway analysis **B.** of a complement of proteins mapped on the surface of MCF10A-KRas^{G12V} and MCF10A-EV cells using global SGM proteomics.

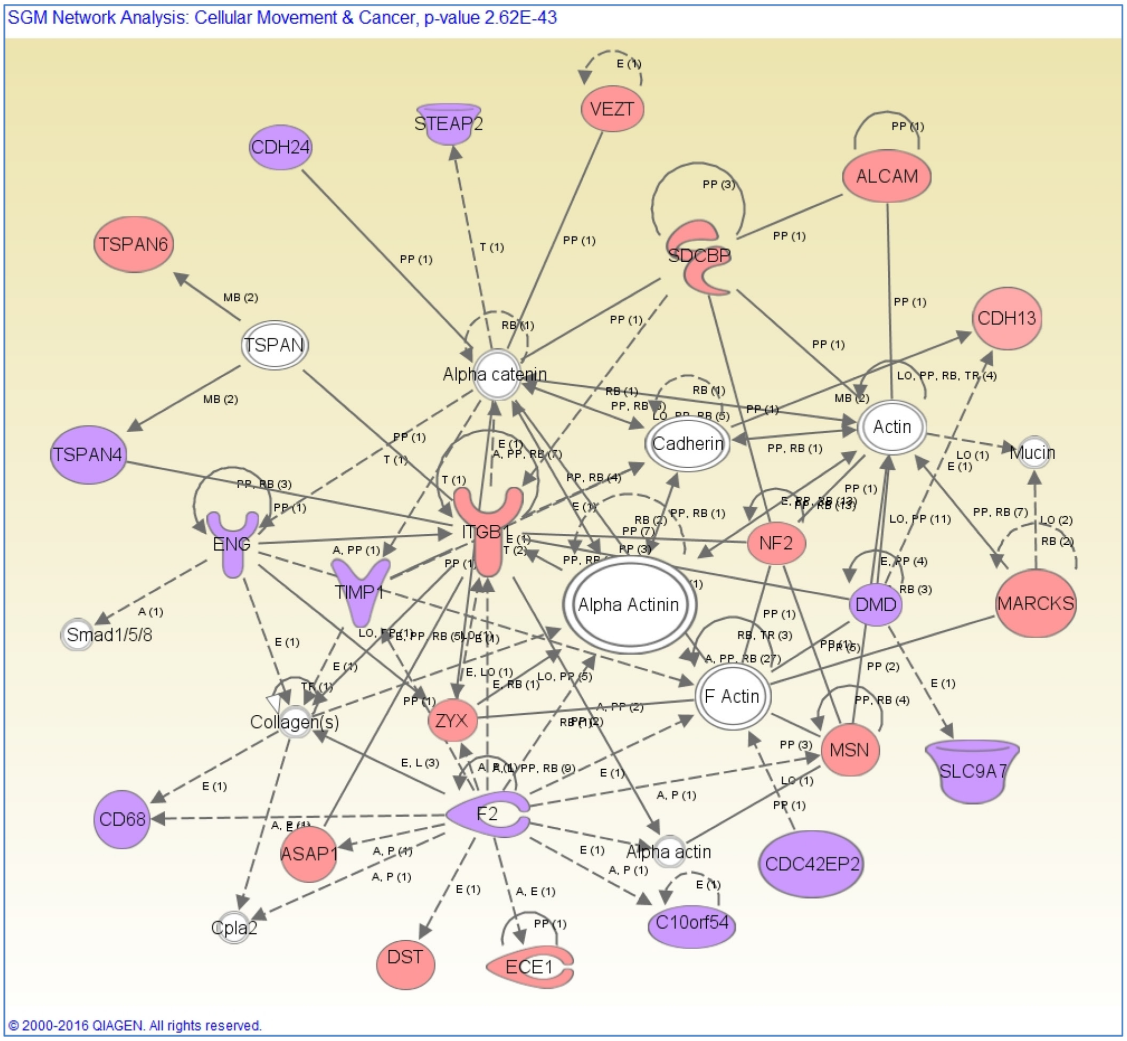

Supplementary Figure S10: IPA ® Network Analysis: Cellular Movement & Cancer, p-value 2.62E-43. Purple color depicts proteins unique to the KRASG12V surface, red color depicts proteins up-regulated on the $KRas_{G12V}$ surface. Solid lines show direct interactions, while dotted lines show indirect protein-protein interactions described in the literature.

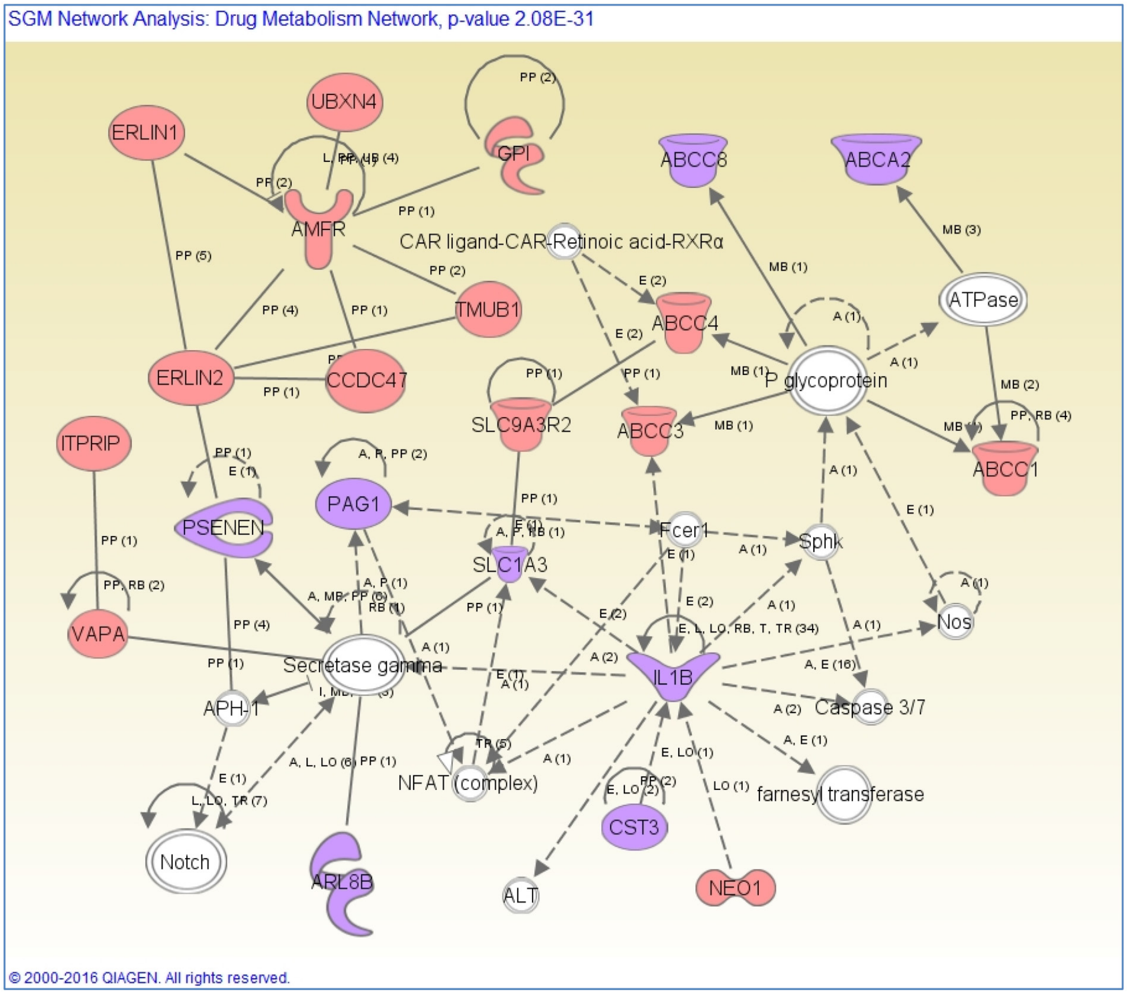

Supplementary Figure S11: IPA ® Network Analysis: Drug Metabolism Network, p-value 9.48E-33. Purple color depicts proteins unique to the KRAS^{G12V} surface, red color depicts proteins up-regulated on the KRas^{G12V} surface. Solid lines show direct interactions, while dotted lines show indirect protein-protein interactions described in the literature.

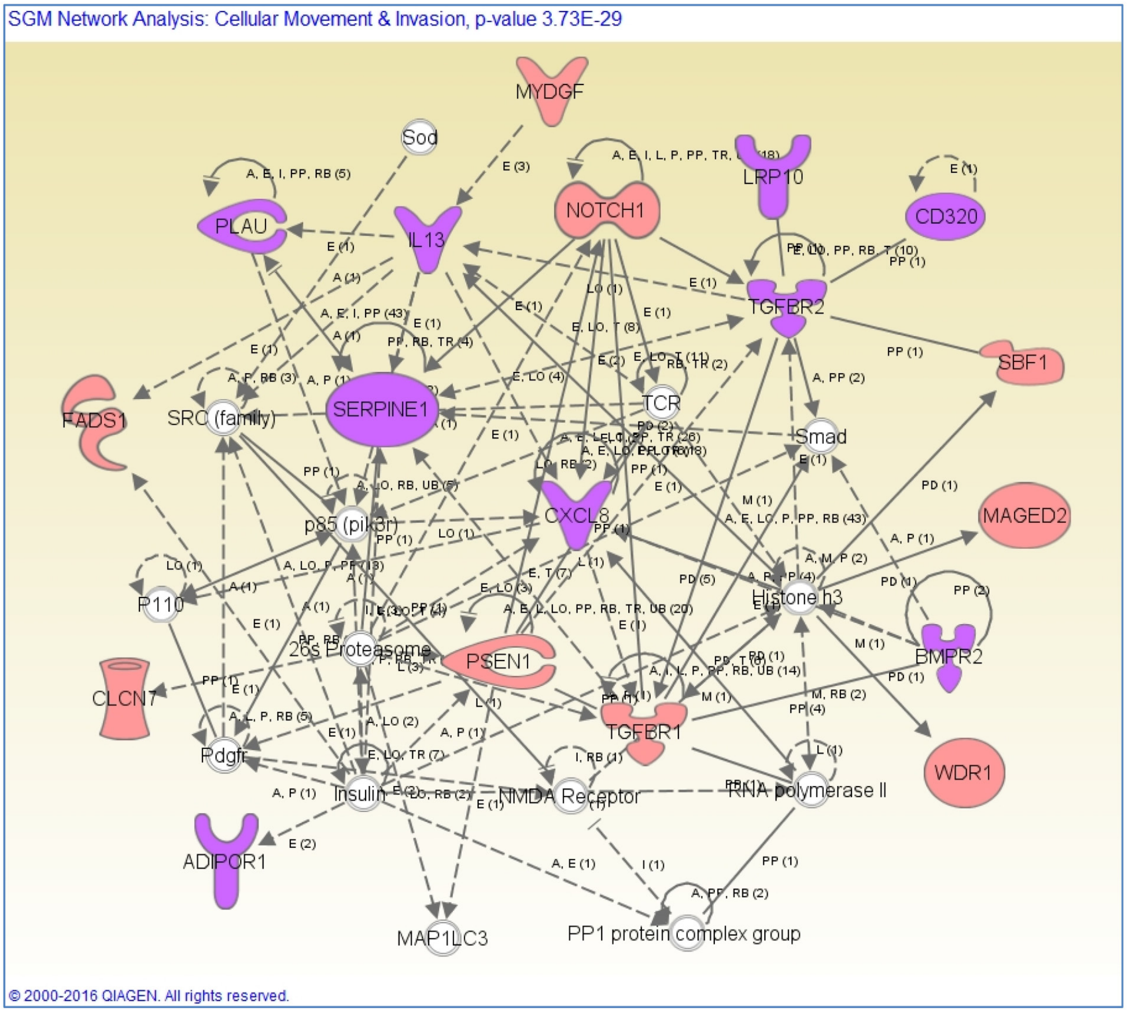

Supplementary Figure S12: IPA ® Network Analysis: Cellular Movement & Invasion, p-value 3.73E-29. Purple color depicts proteins unique to the KRAS^{G12V} surface, red color depicts proteins up-regulated on the KRas^{G12V} surface. Solid lines show direct interactions, while dotted lines show indirect protein-protein interactions described in the literature.

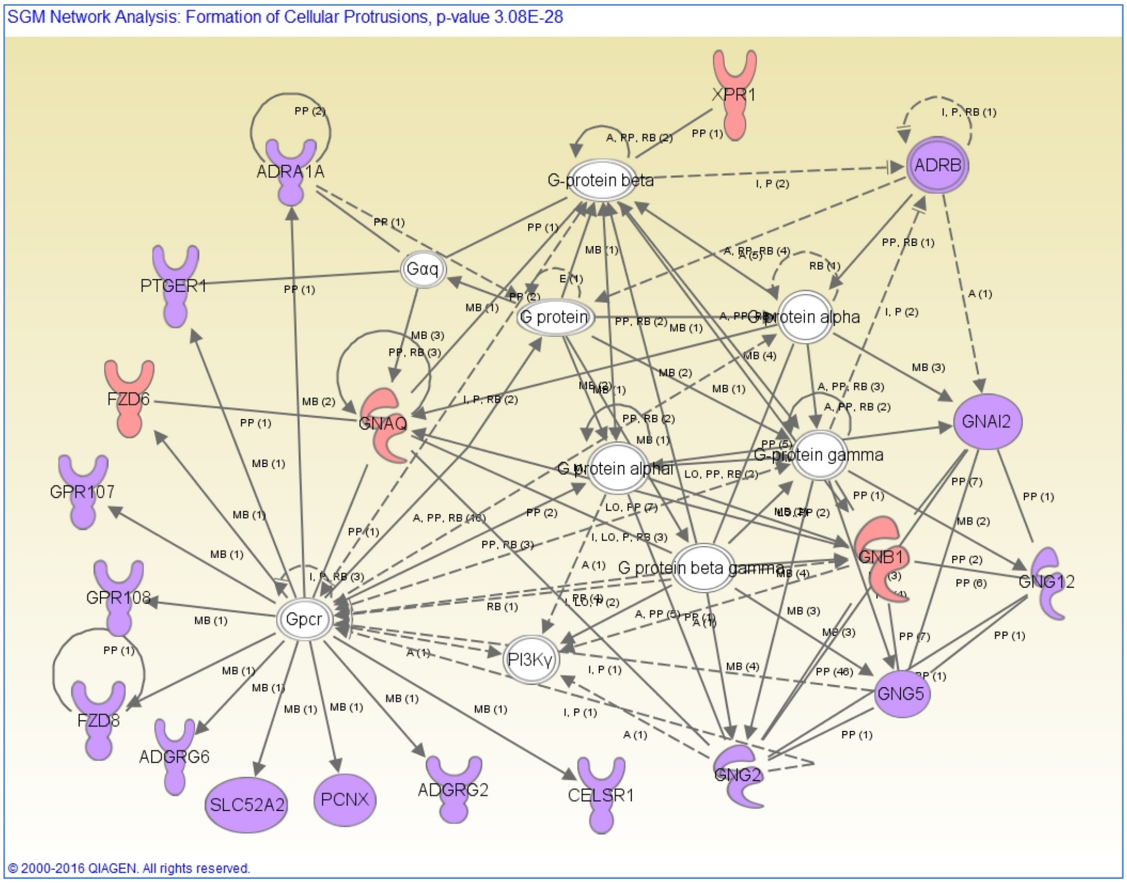

Supplementary Figure S13: IPA ® Network Analysis: Formation of Cellular Protrusions, p-value 3.08E-28. Purple color depicts proteins unique to the $KRas^{G12V}$ surface, red color depicts proteins up-regulated on the $KRas^{G12V}$ surface. Solid lines show direct interactions, while dotted lines show indirect protein-protein interactions described in the literature.

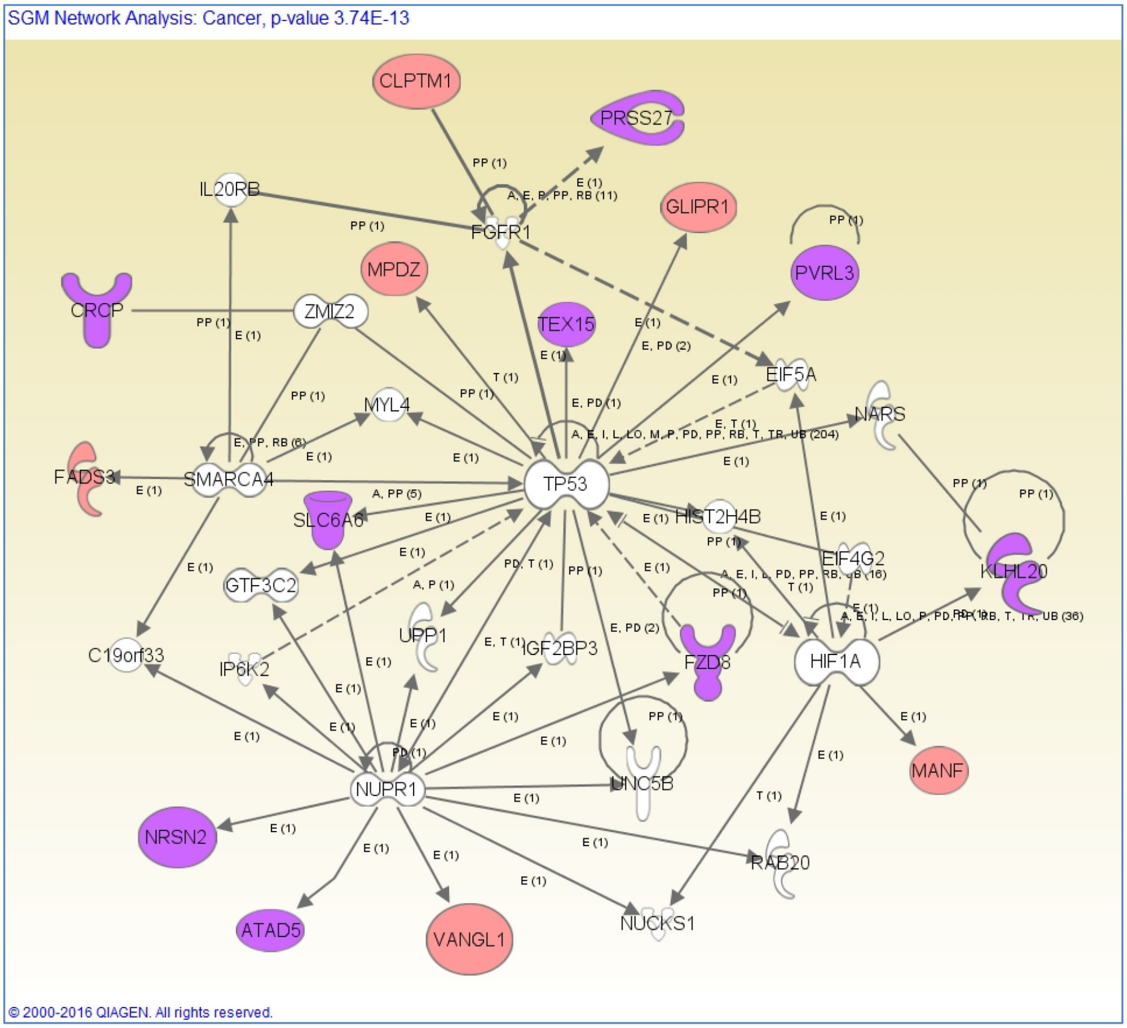

Supplementary Figure S14: IPA ® Network Analysis: Cancer, p-value 3.74E-13. Purple color depicts proteins unique to the $KRas^{G12V}$ surface, red color depicts proteins up-regulated on the $KRas^{G12V}$ surface. Solid lines show direct interactions, while dotted lines show indirect protein-protein interactions described in the literature.

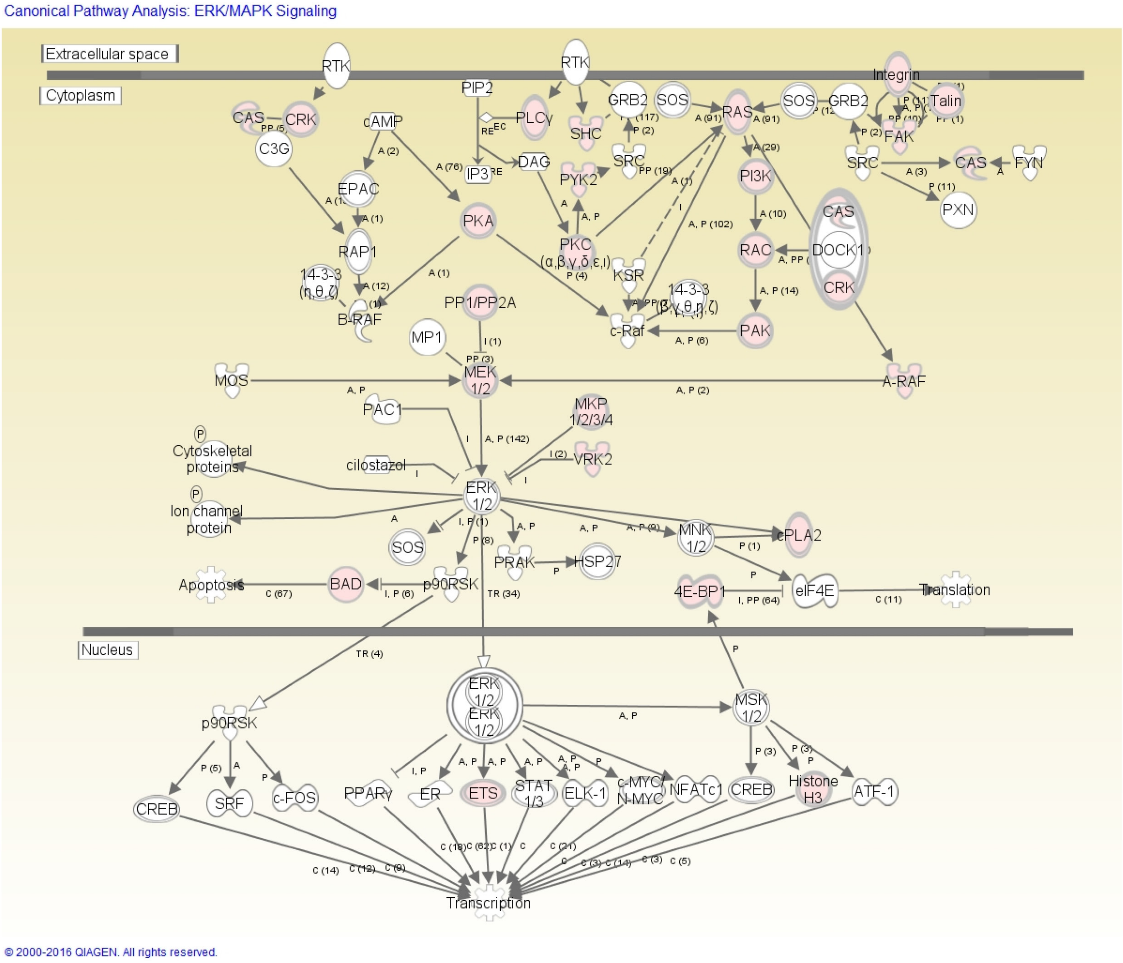

© 2000-2016 QIAGEN. All rights reserved.

Supplementary Figure S15: IPA® Canonical Pathway Analysis: ERK/MAPK pathway – activation z-score 3. 812, p-value 6.81E-04.

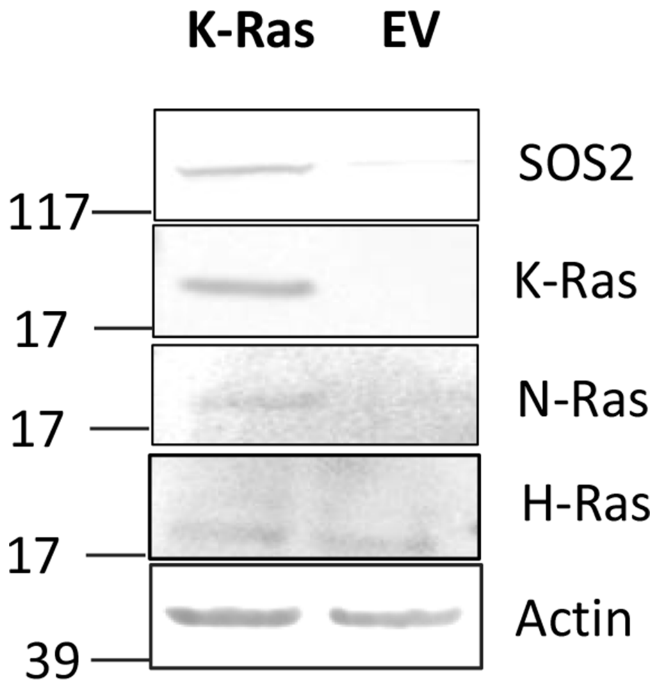

Supplementary Figure S16: ComparativeWB showing cropped images of SOS, K-Ras, N-Ras, and H-Ras in MCF10A-KRas^{G12V} and MCF10A-EV cells.

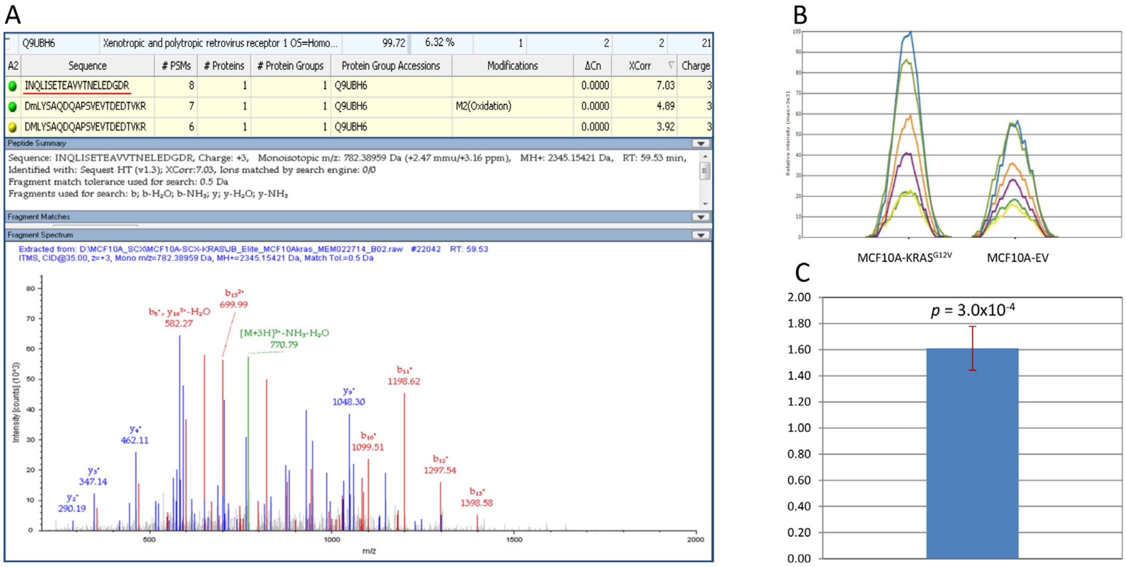

Supplementary Figure S17: A. MS^2 spectrum of the XPR1 peptide INQLISETEAVVTNELEDGDR (underlined in red) acquired in the discovery phase and selected for the synthetic heavy labeled peptide internal standard for the PRM assay development. B. Extracted ion chromatograms of the six most intense fragment ions. Using XPR1 heavy peptide standard Pinpoint calculated normalized $KRas^{G12V}$/EV ratio of 2.27. **C.** Calculated ratio of the ion intensities from extracted ion chromatograms (XICs) and the corresponding p-value for the six XPR1 peptide fragment ions detected by PRM in the membrane preparations of MCF10-$KRas^{G12V}$ and MCF10A-EV.

Supplementary Table S1: (A). N-glycosylated proteins identified on the surface of MCF10A-KRAS^{G12V} cells using targeted CSC proteomics. (B). N-glycosylated peptide identifications in MCF10A-KRAS^{G12V} cells using semitryptic database search and manually validated MS^2 spectra containing NXST N-glycosylation AA motif.

See Supplementary File 1

Supplementary Table S2: (A). N-glycosylated proteins identified on the surface of MCF10A-EV cells using targeted CSC proteomics. (B). Identified N-glycosylated peptides in MCF10A-EV cells using semitryptic database search and manually validated MS^2 spectra containing NXST N-glycosylation AA motif.

See Supplementary File 1

Supplementary Table S3: (A). Cell surface N-glycosylated proteins found unique or S3 (B) significantly upregulated (p-value $\leq$ 0.05) on the surface of MCF10A-KRAS^{G12V} cells using CSC technology.

See Supplementary File 1

Supplementary Table S4: (A). IPA© "Network Analysis" of N-glycosylated proteins found unique or significantly up-regulated (p value$\leq$ 0.05) on the surface of MCF10A-KRAS^{G12V} cells using CSC technology. (B). IPA© "Network Analysis" of proteins found unique or significantly up-regulated (p value$\leq$ 0.05) on the surface of MCF10A-KRAS^{G12V} cells using global SGM proteomics.

See Supplementary File 1

Supplementary Table S5: (A). A list of proteins identified in the membrane fraction of MCF10A-KRAS^{G12V} cells using global SGM proteomics. (B). Identified peptides in the membrane fraction of MCF10A-KRAS^{G12V} cells using SGM proteomics.

See Supplementary File 1

Supplementary Table S6: (A). A list of proteins identified in the membrane fraction of MCF10A-EV cells using global SGM proteomics. (B). Identified peptides in the membrane fraction of MCF10A-EV cells using SGM proteomics.

See Supplementary File 1

Supplementary Table S7: (A). Cell surface proteins found unique or S7 (B) significantly upregulated (p value ≤ 0.05) on the surface of MCF10A-KRAS^{G12V} cells using global SGM proteomics.

See Supplementary File 1

Supplementary Table S8: (A). List of non-redundant proteins found unique or 8 (B) significantly upregulated (p-value ≤ 0.05) on the surface of MCF10A-KRAS^{G12V} cells (B) using both CSC and SGM proteomics.

See Supplementary File 1

Supplementary Table S9: IPA© "Canonical Pathway Analysis" of proteins found unique or significantly upregulated on the surface of MCF10A-KRAS^{G12V} cells using both CSC and SGM proteomics.

See Supplementary File 1

Supplementary Table S10: Constitutive proteins of the Ras pathway (i.e., draw 2.0) identified in MCF10A-KRAS^{G12V} (A) and MCF10A-EV (B) cells.

See Supplementary File 1

Phenotypical cancer cell assays

Boyden chamber migration assays

Culture medium was exchanged for serum-free, supplement-free medium (DMEM/F12) when MCF-10A cells were approximately 60% confluent. After a 24-hour serum deprivation, cells were harvested and counted, and two concentrations of cells (2.5 x 104 and 1.25 x 104) were added to the top of a Boyden chamber with 8 µm pores, in three replicates for each cell density, for each construct. A chemo-tactic gradient was established by adding a chemo-attractant (DMEM/F12 + 5% Horse Serum, 20 ng/ml EGF, 0.5 µg/ml Hydrocortisone, 100 ng/ml Cholera Toxin, and 10 µg/ml Insulin) to the bottom chamber. The cells were allowed to migrate through the 8 µm pores towards the chemo-attractant for 24 hours. Cells were washed, dissociated from the bottom of the Boyden chamber, and incubated with the live cell-labeling dye Calcein-AM (Trevigen, Inc., Gaithersburg, MD) for 1 hour. Fluorescence was measured in a SpectraMax M5 plate reader (Molecular Devices, Sunnyvale, CA) with 485 nm excitation, 538 nm emission, and a 530 nm cutoff filter, as determined by the Calcein-AM emission spectrum. Fluorescence measurements were converted to number of cells using a standard curve established at the beginning of the assay. Percent migration was calculated by averaging the number of cells which migrated towards the chemo-attractant (average of triplicates) at both cell densities after 24 hours, and then averaging the migration percentage for each cell density.

Boyden chamber invasion assays

Culture medium was exchanged for serum-free, supplement-free medium (DMEM/F12) when MCF-10A cells were ~60% confluent. After a 24-hour serum deprivation, cells were harvested, counted, and two concentrations of cells (2.5 x 104 and 1.25 x 104 cells) were added to the top of a Boyden chamber containing a low or high concentration of extracellular matrix (ECM) (Trevigen, Inc., Gaithersburg, MD) overlaid across 8 µm pores, in three replicates for each cell density for each construct. A chemo-tactic gradient was established by adding a chemo-attractant (DMEM/F12 + 5% Horse Serum, 20 ng/ml EGF, 0.5 µg/ml Hydrocortisone, 100 ng/ml Cholera Toxin, and 10 µg/ml Insulin) to the bottom chamber. The cells were allowed to invade and degrade the ECM and then migrate through the 8 µm pores towards the chemo-attractant for 24 hours. Percent invasion was determined as described in migration assay.

Anchorage-independence assays

Iscove's modified Dulbecco's Medium (IMDM; Invitrogen 1200-036) was prepared as a 2x stock in cell culture grade water (Life Technologies, A1287301). To the 2x IMDM the following were added: 20% (v/v) Fetal Bovine Serum, 2 Non-Essential Amino Acids (NEAA; Life Technologies, 1140-050), 200U/mL Penicillin/Streptomycin (Life Technologies, 15140-122) and 0.6% (w/v) sodium bicarbonate (Sigma-Aldrich, S5761-500G). This solution was passed through a 0.20µm filter (Nalgene, 66401221) and hereafter will be referred to as 2x IMDM Complete. The 2x IMDM Complete media was further diluted in equal parts with cell culture grade water to make 1x IMDM Complete media.

Polyhydroxyethylmethacrylate- coated (Poly-HEMA; Sigma-Aldrich, P3932) plates were prepared as follows. First 1.64g of Poly-HEMA was dissolved in 10mL methanol at 55°C for 3 hours, then further diluted with 40mL Ethanol (95% solution). The Poly-HEMA solution was used to coat each well of a clear, flat bottom 96 well plate with 10µL. Plates were dried at ambient temperature for up to 72 hours. Coated plates were UV-treated for sterility and stored until use. Stock aliquots of 1.2% (w/v) Agarose II (low melting temperature agar; G-Biosciences, RC-008) were created in cell culture grade water. Aliquots were stored at 4°C for up to one week for use.

Cells were harvested from cell-specific growth conditions during log phase growth. After harvest and phosphate buffered saline, pH 7.4 (PBS) wash, cells were re-suspended to create cell stocks at 4.0E5 cells/mL (high density) and 0.5E5 cells/mL (low density) in PBS alongside a cell-free (PBS) control. Cell suspensions (or PBS control) were further diluted 1-to-10 by volume in 1x IMDM Complete for Anchorage-Dependent and Poly-HEMA conditions, or in 2x IMDM Complete for Soft-agar conditions. Anchorage-Dependent and poly-HEMA plates were loaded in quintuplicate with 100µL of high density and low density dilutions alongside the cell-free control in duplicate. Plates were incubated for 7 days at 37°C and 5% CO2.

Soft-agar plates were created by dissolving a 1.2% agar aliquot at 75°C for 30 minutes, and then placed at 37°C for 1 hour. Soft-agar plates consist of three layers, the bottom and top layer are created with equal parts 2x IMDM and 1.2% agar, while the middle layer is created with agar, cells and 2x IMDM. Inside a microcentrifuge tube, 25µL 1.2% agar and 25µL 2x IMDM Complete were mixed then immediately transferred to each well of a 96 well plate. Plates were placed at 4°C for 5 minutes to allow agar to solidify bottom layer. In another microcentrifuge

tube, 100µL of cell suspensions (or cell-free control) in 2x IMDM complete were diluted using 200µL 2x IMDM and 300µL 1.2% agar. From each, 60uL were loaded in quintuplicate or 60µL of cell-free control in duplicate. Plates were placed at 4°C for 5 minutes to allow agar to solidify. Steps taken to create the bottom layer were repeated to create the top layer. Plates were placed at 4°C for 5 minutes to solidify agar, then placed at 37°C, 5% CO_2 for 7 days. On Day 7, every plate received 16µL of AlamarBlue (Invitrogen, DAL1025) per well. Plates were incubated for 3 hours at 37°C and 5%CO_2 before reading (excitation 530 nm & emission, 590nm).
